# Understanding the complementarity and plasticity of antibody–antigen interfaces

**DOI:** 10.1093/bioinformatics/btad392

**Published:** 2023-06-29

**Authors:** Yoochan Myung, Douglas E V Pires, David B Ascher

**Affiliations:** Structural Biology and Bioinformatics, Department of Biochemistry and Pharmacology, University of Melbourne, Melbourne, VIC 3010, Australia; Computational Biology and Clinical Informatics, Baker Heart and Diabetes Institute, Melbourne, VIC 3044, Australia; Systems and Computational Biology, Bio21 Institute, University of Melbourne, Melbourne, VIC 3052, Australia; School of Chemistry and Molecular Biosciences, University of Queensland, St Lucia, QLD 4072, Australia; Computational Biology and Clinical Informatics, Baker Heart and Diabetes Institute, Melbourne, VIC 3044, Australia; Systems and Computational Biology, Bio21 Institute, University of Melbourne, Melbourne, VIC 3052, Australia; School of Computing and Information Systems, University of Melbourne, Melbourne, VIC 3010, Australia; Structural Biology and Bioinformatics, Department of Biochemistry and Pharmacology, University of Melbourne, Melbourne, VIC 3010, Australia; Computational Biology and Clinical Informatics, Baker Heart and Diabetes Institute, Melbourne, VIC 3044, Australia; Systems and Computational Biology, Bio21 Institute, University of Melbourne, Melbourne, VIC 3052, Australia; School of Chemistry and Molecular Biosciences, University of Queensland, St Lucia, QLD 4072, Australia

## Abstract

**Motivation:**

While antibodies have been ground-breaking therapeutic agents, the structural determinants for antibody binding specificity remain to be fully elucidated, which is compounded by the virtually unlimited repertoire of antigens they can recognize. Here, we have explored the structural landscapes of antibody–antigen interfaces to identify the structural determinants driving target recognition by assessing concavity and interatomic interactions.

**Results:**

We found that complementarity-determining regions utilized deeper concavity with their longer H3 loops, especially H3 loops of nanobody showing the deepest use of concavity. Of all amino acid residues found in complementarity-determining regions, tryptophan used deeper concavity, especially in nanobodies, making it suitable for leveraging concave antigen surfaces. Similarly, antigens utilized arginine to bind to deeper pockets of the antibody surface. Our findings fill a gap in knowledge about the antibody specificity, binding affinity, and the nature of antibody–antigen interface features, which will lead to a better understanding of how antibodies can be more effective to target druggable sites on antigen surfaces.

**Availability and implementation:**

The data and scripts are available at: https://github.com/YoochanMyung/scripts.

## 1 Introduction

Over the past decades, advances in immunotherapy have been revolutionizing targeted development, particularly for cancer treatment (Zahavi and Weiner 2020). Immunotherapy utilizes hosts’ immune systems to combat diseases by minimizing the possible adverse effects from using foreign molecules. Immunotherapeutic agents including antibody therapeutics have high target binding specificity and affinity, which also determines the efficacy and safety of the treatments. Based on their modes of action, antibodies provide prompt and temporary immunity which make them even more suitable for a variety of applications such as immunosuppressed patients and organ transplant recipients than other therapeutic agents.

Antibodies typically consist of a heavy chain and a light chain component. The variable domains of heavy (V_H_) and light (V_L_) chains are responsible for antigen binding. Light chains have a single constant region, which dimerizes with the first of three heavy chain constant regions to form a fragment antigen-binding (Fab). Fabs with heavy and light chain components are known as V_H_V_L_ Fab. Similar to Fab, single-chain fragment variable (scFv) consists of V_H_ and V_L_ of Fab coupled by a linker but not by constant regions (Fc). However, nanobodies have single heavy chain Fab regions, V_HH_, known to have lower cost of production, high affinity for antigens, relatively low molecular weight, and cell and blood-brain barrier-penetrating potential in comparison with Fab and scFv antibodies (Li *et al.* 2012, Muyldermans 2013, Chan *et al.* 2015, Chan *et al.* 2016). Anecdotally, some V_HH_ antibodies have been identified as having the potential to bind into deeper pockets or clefts than VHVL antibodies and globular protein interactions (Desmyter *et al.* 1996, Desmyter *et al.* 2002, De Genst *et al.* 2006, Rouet *et al.* 2015, Rodrigues *et al.* 2022). However, there are only limited explanations of features that can account for the ability of single heavy chain antibody to bind to concave surfaces of antigens, which are generally inaccessible by two-chain antibodies. This emphasizes the necessity of various levels of analysis for understanding antibody binding modes.

Antibodies have the highly variable regions of heavy and light chains which are known as Complementarity-Determining Regions (CDRs). CDRs confer high target binding affinity and specificity, generated through clonal selection of immune B-cells that have produced antibodies with varying affinities by both V(D)J recombination and somatic hypermutation of hypervariable DNA regions corresponding to CDRs (MacCallum *et al.* 1996).

Notably, a key area of interest with respect to use of antibodies as drugs is minimization of binding interfaces and identification of the key features that drive their potent molecular recognition. While many studies examined sequence (Soga *et al.* 2010, Kunik and Ofran 2013, Xin *et al.* 2018) and structural characteristics (Ramaraj *et al.* 2012, Peng *et al.* 2014, Nguyen *et al.* 2017, Daberdaku and Ferrari 2019) of antibody–antigen interfaces, their key features determining antigen recognition are not well understood, with most studies limited to assessing residue propensity and presence of hotspots. This is further convoluted by the diversity of proteins recognized by a finite repertorie of antibodies. Regarding residue prevalence in interfaces, arginine is the most abundant amino acid in protein–protein interfaces, capable of establishing cation-π and hydrogen bond interactions with aromatic residues such as tyrosine and tryptophan (Crowley and Golovin 2005). Alternatively, the analysis of the 53 antibody–antigen complexes (Ramaraj *et al.* 2012) identified tyrosine and lysine as the most abundant in paratope and epitope surfaces respectively, although aromatic residues had the highest propensity in antibody–antigen interfaces. Aromatic residues in antibodies, especially tyrosine and tryptophan, contribute to the half of hotspots (ΔΔG > 1 Kcal/mol) (Dall'Acqua *et al.* 1996, Bostrom *et al.* 2009, Pires and Ascher 2016, Myung *et al.* 2020, Myung *et al.* 2020), signifying the binding specificity and affinity of antibodies can be associated with the geometry and noncovalent interactions of aromatic residues (Peng *et al.* 2014). On the other hand, distinctive features of epitopes were not observed compared to non-antigen surfaces that can affect antibody binding. Collectively, this can explain why the performance of current conformational epitope predictions are more limited (Peng *et al.* 2014) than that of CDR modelling.

In this article, we further explored the nature of antibody–protein interfaces for V_H_V_L_ and V_HH_ antibodies, specifically with respect to use of concavity and molecular interactions. We tested the hypothesis that antibody concavity is correlated with CDR length, finding a weak correlation for Fab and V_HH_ CDR H3 regions, and a moderate correlation for scFv CDR H3. We found significant relationships between tryptophan in V_HH_ CDR H3 and use of concavity, but statistically insignificant relationships between CDR residue position and use of concavity. Similarly, analysis of interatomic interactions between V_H_V_L_ and V_HH_ antibodies did not reveal significant differences between antibody types. We found that CDR use of concavity was reciprocated by antigen residues binding into their antibodies, but more consistently so for Fab and V_HH_ antibody–antigen interfaces than for scFv antibody–antigen interfaces. Based on our analysis, the ability of nanobodies to target druggable sites on antigen surfaces could be explained by the propensity of specific amino acids and their positions in CDRs, which can help guide the development of an antibody targeting a traditional small-molecule binding site.

## 2 Materials and methods

### 2.1 Dataset

We utilized the SAbDab database (Dunbar *et al.* 2014) as the basis for determining antibody complex structures from the wwPDB. As of November 2021, 4915 antibody structures were available and we filtered the following structures: (i) 409 unbound antibodies and non-protein/peptide-antibody complexes, (ii) 222 redundant information, (iii) 1851 low-resolution (>3 Å) complexes, (iv) 3 structures with UNK residues and (v) 13 Fragment light chain dimer (Fll) complexes. We retained 2417 antibody–antigen complexes consisting of 1753 Fab, 73 scFv, and 591 V_HH_ structures for the further data redundancy assessment.

**Figure 1.**
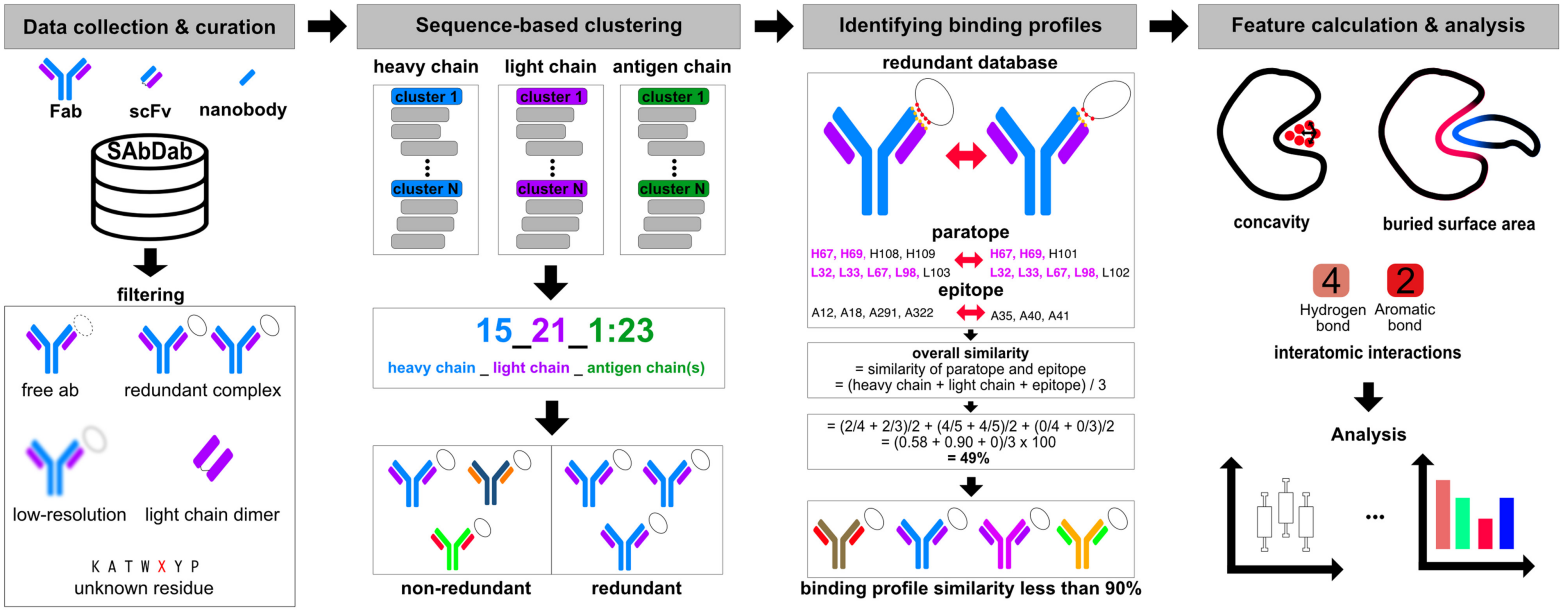
Workflow of antibody–antigen landscape analysis. Collected Fab-, scFv-, and V_HH_-antigen complexes from SAbDab are filtered if there is a sole antibody, redundant complex, low-resolution (>3 Å), light-chain only antibody/complex and antibody–antigen complex with unknown residues (UNK). To get the information of nonredundant antibody–antigen interfaces, the filtered complexes are further processed with ‘sequence-based clustering’ and ‘interface structure-based similarity scoring’. Using CD-HIT, each chain of antibody–antigen complexes is clustered with the threshold of 0.95 separately and filtered by their new cluster labels. Since the sequence-based clustering cannot distinguish antibody–antigen complexes that have the same sequences but different binding sites, we further consider the information of the paratope and epitope residues. The redundancy assessment using paratope and epitope residues is done in a pairwise manner by checking across all possible combinations of pairs of antibody–antigen complexes. The interface similarity of each pair of antibody–antigen complexes is calculated by two aspects (one from the first complex and another from the other complex) and then their scores are averaged to get an overall similarity score for the complex. In this way, those complexes that are considered as ‘redundant’ in the earlier clustering approach can be kept in the nonredundant database if redundant complexes have scores of interface similarity <90%. Finally, the concavity (*R*_inaccess_), buried surface area, and interatomic interactions are calculated on the nonredundant complexes and analysed.

In order to avoid redundant antibody–antigen complex information in the analysis, we implemented a new approach by combining sequence-alignment-based clustering and interface-based structure comparison methods (Fig. 1). The major reason for applying two methods is that the conventional sequence-based clustering is not ideal in the case of identifying antibodies that bind to the same antigens but on different binding sites. To do that, firstly, we clustered heavy, light, and antigen chains individually with the sequence identity threshold of 0.95 using CD-HIT (Fu *et al.* 2012), then labelled all structures according to their cluster numbers. For instance, if an antibody–antigen complex includes one heavy (cluster #15) and one light (cluster #21) and two antigen chains (cluster #1 and #23), the complex was labelled as ‘15_21_1:23’. From CD-HIT clustering, we sorted 2417 antibody–antigen complexes into 670 nonredundant and 1217 redundant complexes.

To determine antibody–antigen complexes which have 95% sequence similarity but have different binding/interface profiles, the paratope, and epitope residues of the 1217 redundant complexes were identified using Pymol (v.2.3.4) and compared based on IMGT (Lefranc *et al.* 2005) numbering. At last, the 553 redundant complexes which shared <90% of epitope and paratope residues were reconsidered as nonredundant complexes; thus, we constructed a database of 1223 antibody–antigen complexes including 835 Fab, 73 scFv, and 312 V_HH_ structures (Supplementary Data). The overall workflow of the dataset preparation is described in Fig. 1.

### 2.2 Concavity calculation

The concavity of antibody–antigen interface residues was measured using Ghecom (Kawabata 2010). The *R*_inaccess_ value of Ghecom represents the smallest spherical probe size by varying its radius from 2.5 to 10.5 Å that is unable to enter a space on a partner protein’s surface. Thus, attributing a numerical measure of the extent to which a point in space, such as an atom, makes use of concavity, and summarized per residue by averaging the atomic *R*_inaccess_ values in residues. To analyse the deepest concavity usage of antibody–antigen interfaces, the minimum (deepest bound) atomic values of *R*_inaccess_ in residues were used.

### 2.3 Interatomic interactions

Interatomic interactions that occurred at antibody–antigen interfaces were measured using Arpeggio (Jubb *et al.* 2017), after normalizing interface size of each complex by comparing interactions per 100 Å BSA (Buried Surface Area). To minimize the calculation costs, only residues of paratope and epitope where the interface residues were found within 5 Å to their binding partners were considered.

### 2.4 Buried surface area calculation

Buried surface area (BSA) between paratope and epitope can be calculated by the following equation (1) using FreeSASA (Mitternacht 2016). This equation measures the solvent accessible surface area (SASA) difference between the sum of free antibody (SASA_Ab_) and antigen (SASA_Ag_) chains and their bound form (SASA_Complex_). The reference information of atomic radii was based on NACCESS (Hubbard and Thornton 1993) but C_alpha_ was not treated as ‘side-chain’ as in NACCESS.


(1) $${\text{BSA}}_{\text{Ab}:\text{Ag}}=\left({\text{SASA}}_{\text{Ab}}+{\;\text{SASA}}_{\text{Ag}}\right)\;–{\;\text{SASA}}_{\text{Complex}}$$

### 2.5 Statistical analysis

We used one-way analysis of variance (ANOVA) to determine the statistical significance of differences of use of concavity among different groups (e.g. amino acid, CDR residue position, and CDR length) using R-4.0.3. Further, the Tukey Honest Significant Differences (Tukey HSD) test was conducted to compare the pairwise means of the ANOVA groups.

## 3 Results

### 3.1 Diversity of CDR length

To understand the relationships between the size of CDRs and their use of concavities in antibody binding, the length of CDRs in Fab, scFv, and V_HH_ antibody–antigen complexes was determined based on IMGT (Giudicelli *et al.* 2003) annotation. The distribution of CDR lengths (Supplementary Fig. S1) in the nonredundant antibody dataset was statistically assessed via the analysis of variance (ANOVA). The results showed that V_HH_-H3 has on average 15 amino acids showing the mean value was 15.4 which is longer than any other kind of CDR, followed by Fab-H3 and scFv-H3 (Supplementary Table S1). Compared to CDRs Fab/V_HH_/scFv-H3 and V_HH_-H1 which have the variance of CDR length ranges between 15 and 21, the variance of CDR length in others is lower showing the maximum range of 11. Overall, all CDR-H3s have significantly longer CDR length averaged from between 11 to 15 amino acids, which implies a possible advantage of using long CDRs to recognize various antigens.

### 3.2 Use of concavity by CDRs

The use of concavity (*R*_inaccess_), which describes ‘how deep a residue binds into a partner surface’, was measured with the different size of Ghecom probe size (Supplementary Fig. S2A) on each atom of antibody–antigen interface residues. To get a residue-level *R*_inaccess_, the deepest atom-level *R*_inaccess_ value was used for residues because averaging atom-level *R*_inaccess_ values of each residue can underestimate the deep use of concavity mostly by side-chain atoms. For example, the tryptophan side chain group has *R*_inaccess_ values in the range of 2.87–8.31 Å (Supplementary Fig. S2B), and the *R*_inaccess_ of 2.87 Å (the deepest use of concavity) is used for the residue. Then, for the analysis of ‘average use of concavity’, the *R*_inaccess_ values of interface residues were averaged based on CDR region or amino acid. On the contrary, a residue with the deepest *R*_inaccess_ on respective CDR region or amino acid was selected for each antibody–antigen complex, and then the average *R*_inaccess_ over all complexes was taken to represent the deepest use of concavity. Simply, the value of use of concavity close to 10.5 Å, equal to the largest probe size, represents an atom binding towards a flat plane, while smaller *R*_inaccess_ indicates an atom binding towards a more concave target surface.

**Figure 2.**
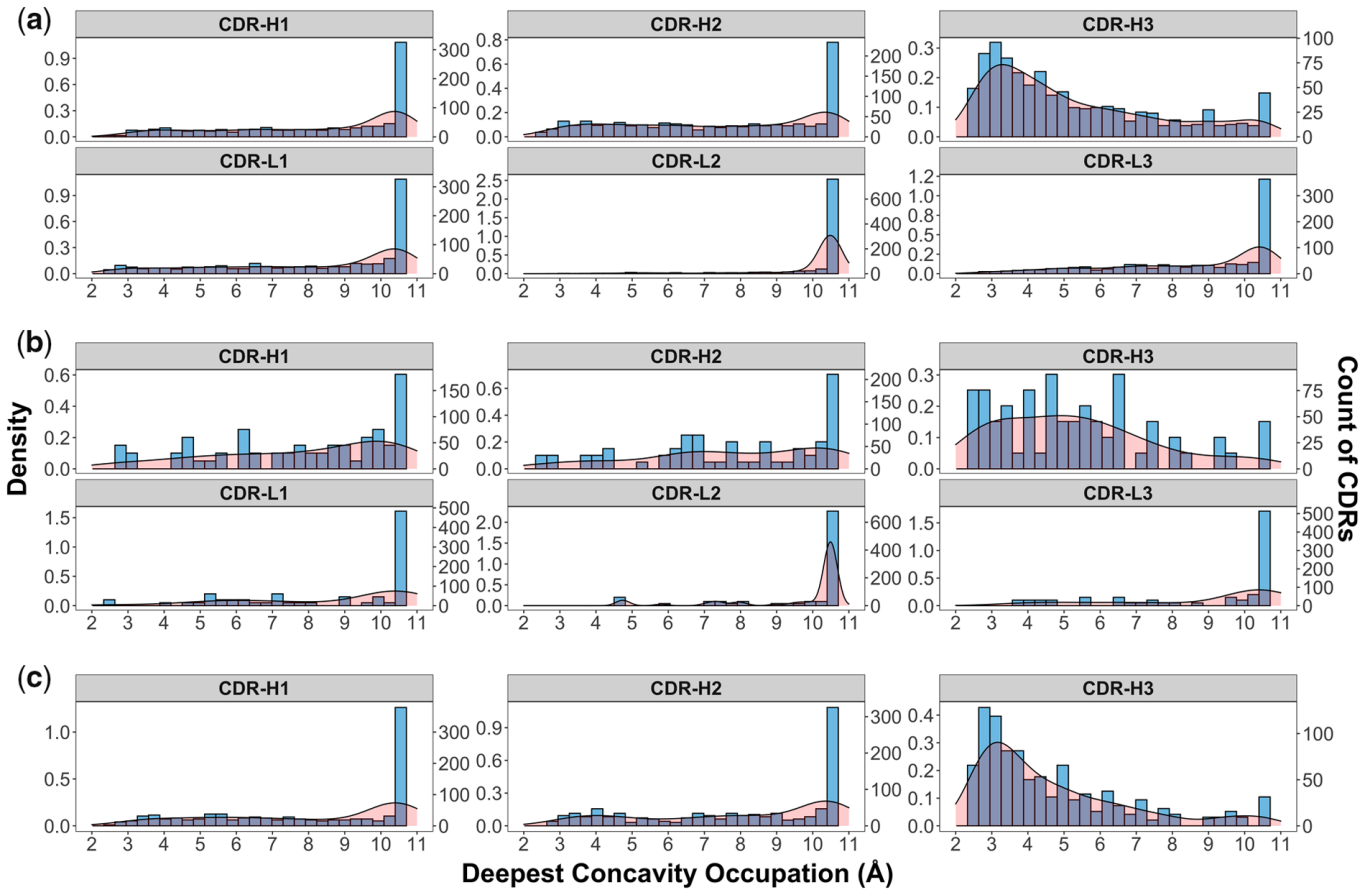
Histogram distribution of deepest use of concavity of CDR regions. Deepest concavity distributions for CDRs in Fab (a), scFv (b), and V_HH_ (c) antibodies. Concavity is as measured by Ghecom, representing the smallest spherical probe size that was able to enter a space around the partner protein's surface (where smaller values represent deeper binding). Concavity was assigned to CDR atoms and the smallest value (deepest protrusion into antigen) was measured for each CDR. Plots are faceted by CDR designation. The Density (left *Y*-axis) and Count of CDRs (right *Y*-axis) are shown in the red shaded curve and blue shaded bar, respectively.

We analysed the extent to which CDRs used concavity when binding to antigens illustrating how Fab, scFv, and nanobodies CDRs used concavity by at deepest (Fig. 2) and on average (Supplementary Fig. S3), respectively. Statistical analysis of these distributions indicated that all CDR H3 regions used deeper concavity on average than any other CDR type (Supplementary Table S2), albeit with only a small difference in magnitude of concavity showing 1.3 Å < 10.5 Å value equating to flatness (no use of concavity). Compared to other H3 CDRs, V_HH_-H3 showed the average concavity of 9.18 Å and the range (difference between maximum and minimum values) of 4.5 Å representing V_HH_-H3 utilizes significantly deeper and more malleable concavity for antibody binding.

A much greater range of concavity was used at the deepest points of CDRs as compared to use on average. H3 CDRs bound at their deepest in more concave binding modes than any other CDR type by 2.1 and 5.3 Å (Supplementary Table S3). While they were not statistically significantly different from each other in their deepest use of concavity, ANOVA groupings overlapped to varying degrees between all other CDR types, with V_HH_-H2, scFv-H2, and CDRs L1, L3, and H1 all being statistically insignificantly different in their deepest use of concavity, which averaged from between 7.7 and 8.9 Å [flat to approaching shallow groove (Kawabata 2019)]. Overall, V_HH_-H3 showed the deepest use of concavity both on average and at deepest achieving 9.18 and 4.66 Å respectively, implying possible benefits of V_HH_ in recognizing shallower pockets and grooves of target surfaces over V_H_V_L_-H3s.

### 3.3 Relationship between CDR length and use of concavity

**Figure 3.**
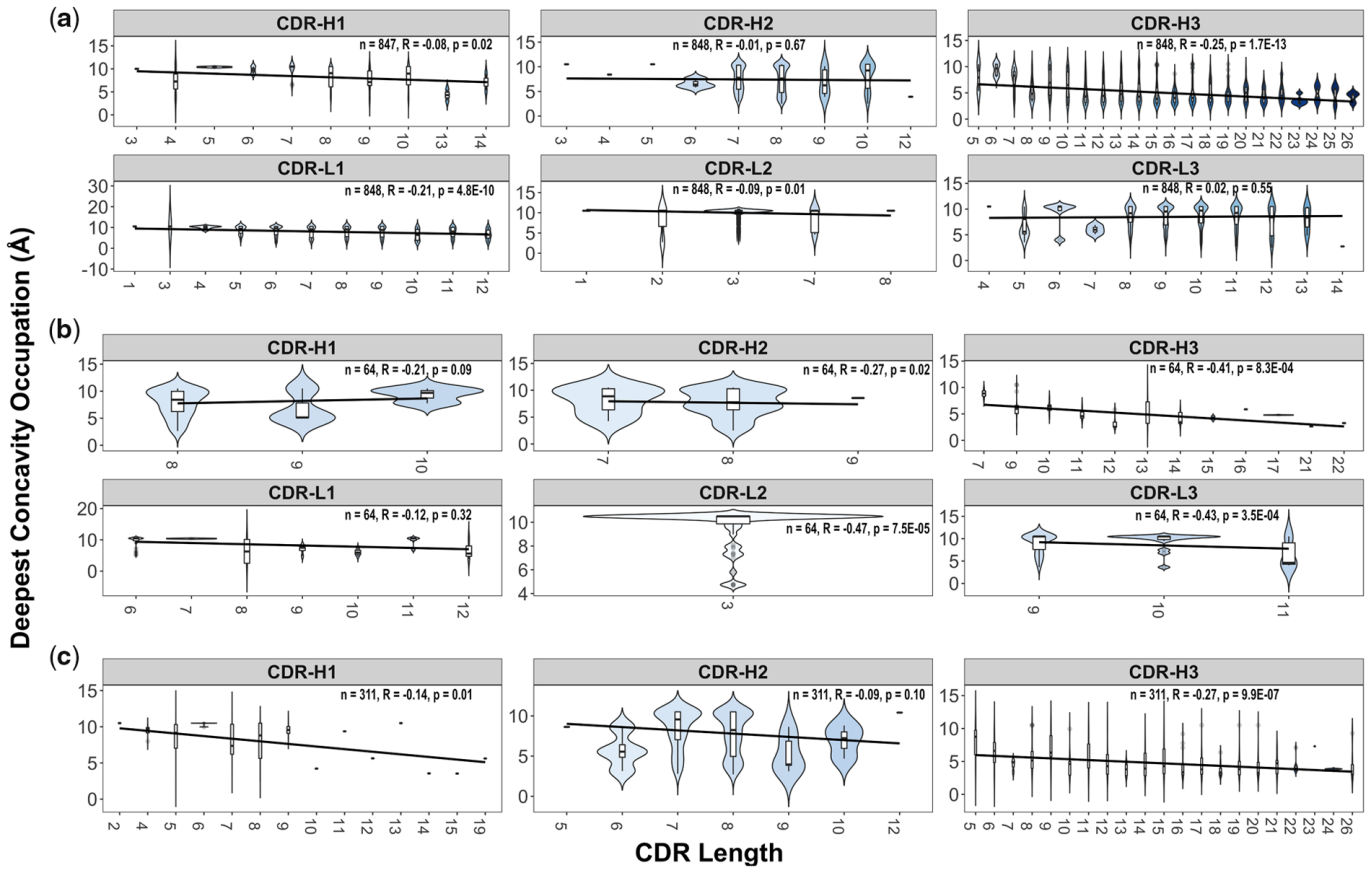
Boxplot distributions and linear correlations illustrating the relationship between CDR length and use of concavity for antibody CDRs. CDR length-concavity relationships are shown for Fab (a), scFv (b), and V_HH_ (c) antibodies. Linear model fitting is shown in slopes.

Anecdotal evidence has suggested a link between antibody CDR length and use of concavity, in particular with respect to V_HH_ antibodies (Desmyter *et al.* 1996, Desmyter *et al.* 2002, De Genst *et al.* 2006, Rouet *et al.* 2015). By comparing CDR length and use of concavity (Fig. 3), we found that CDR use of concavity at deepest was negatively correlated with CDR length for most CDRs, but significantly on CDR H3s. While these correlations were statistically significant (*P* < 0.05), they were weak in magnitude for Fab-H3 and V_HH_-H3, and moderate in magnitude for scFv-H3. Similarly, CDR-H1, H2, L2, L3 of scFv showed weak to moderate correlations, but they have only one to three subgroups of CDR lengths, which is inappropriate to assess the relationship between CDR length and use of concavity. All other CDRs were not significantly correlated with use of concavity. Thus, the three types of antibodies can use their longer H3 CDRs to bind to concave antigen surfaces in general.

### 3.4 Relationship between CDR residue position and use of concavity

Canonical CDR numbering systems such as the IMGT system (Lefranc *et al.* 2005) allow comparison of properties of CDRs between different antibodies, regardless of their target antigen. We used IMGT derived CDR structures from SAbDab to understand how CDR residue concavity use on antigen binding varied with residue CDR position. Most CDR residue positions utilized little or no concavity, but CDR H3s showed a greater tendency for concavity in positions of 110-111C and 112C of Fab-H3, 109-111A and 112-112A of scFv-H3 and 109-111A of V_HH_-H3 (Supplementary Fig. S4), where those residues showed use of concavity <10.5 Å and the number of observations was more than 10% for each antibody group. If we compare those residues that have more than half of their total data points observed, position 110 of CDR-H3 showed the deepest use of concavity across three types of antibodies. This indicates that position 110 of V_H_V_L_ and V_HH_ can be important to recognize shallow grooves of antigen surfaces. While these residues exhibited a tendency to make greater use of concavity compared to other CDR residue positions (Supplementary Fig. S4), ANOVA indicated that these distribution differences to use of concavity at other residue positions were not statistically significant (Supplementary Table S4).

### 3.5 Amino acid concavity preferences for CDRs

**Figure 4.**
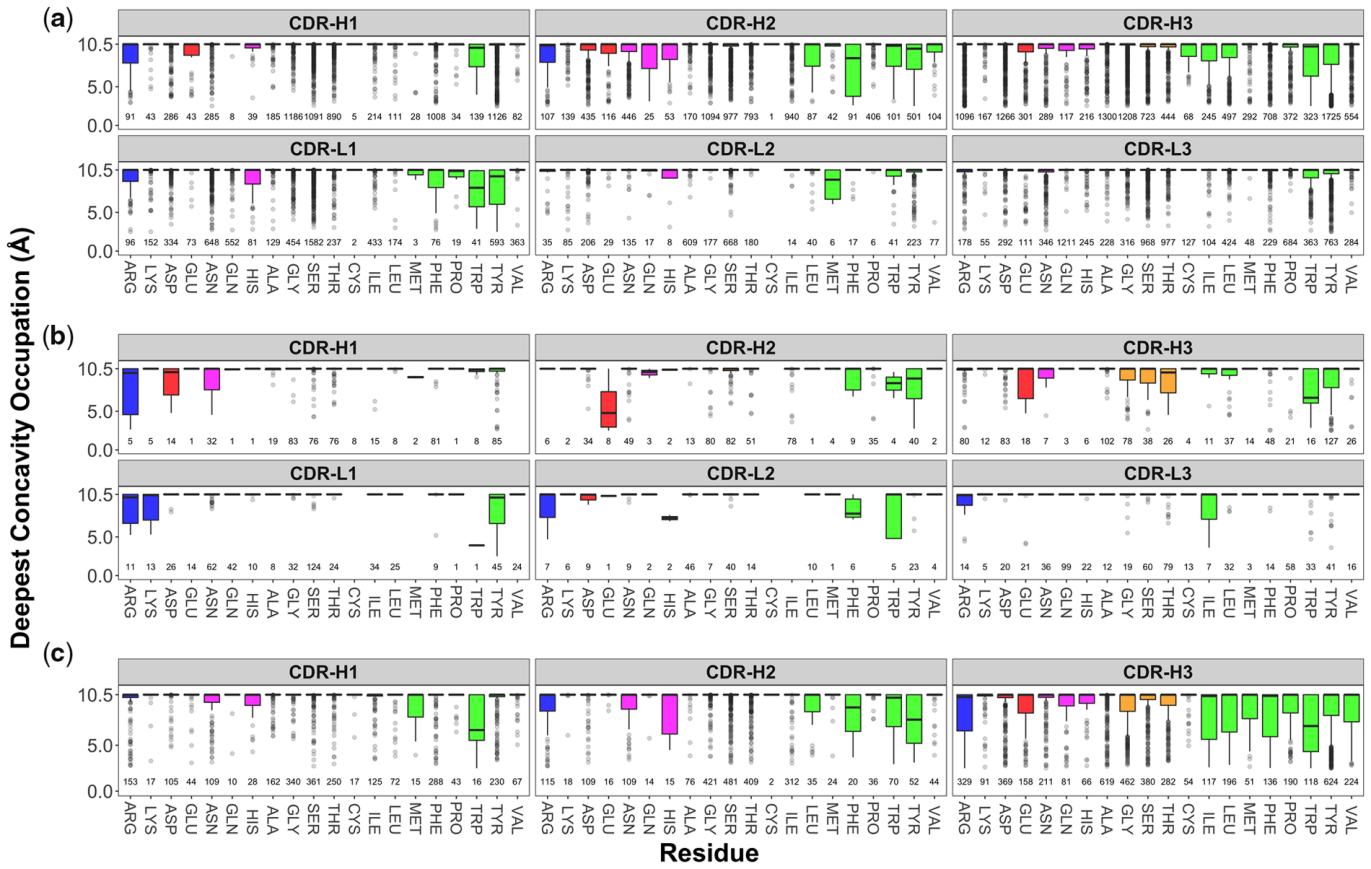
Boxplot distribution of antibody CDR residue use of concavity by amino acid in Fab (a), scFv (b), and V_HH_ (c) antibodies. Residue distributions are coloured using a modified version of the Lesk colour scheme (positively charged = blue, negatively charged = red, polar = magenta, small non-polar = orange, hydrophobic = green).

In addition to understanding use of concavity by antibody CDRs through different sequence-based positioning, we investigated how different amino acids in CDRs make use of concavity. While the majority of amino acids in most CDRs did not overly utilize concavity, several amino acids in different CDR environments were skewed towards using concavity, including tryptophan in V_HH_-H3, V_HH_-H1, scFv-H3, Fab-L1, and tyrosine in CDR H2s (Fig. 4). Compared to other aromatic residues, phenylalanine and tyrosine, tryptophan has a much lower propensity in CDRs (Supplementary Fig. S9), but it can greatly contribute to use of concavity especially for both V_HH_ and V_H_V_L_ (Fig. 4). While these residues had lower concavity values on average than other residues (thus making greater use of concavity), ANOVA indicated that these differences were statistically insignificant (Supplementary Table S5).

### 3.6 Use of concavity by antigens

In addition to assessing how antibody CDRs made use of concavity in antigen binding sites, we tested the hypothesis that antigens make use of concavity formed by antibodies showing the degrees of concavity used by antibody binding interfaces of antigens (Supplementary Fig. S5). Antigens did not make use of concavity on average when binding into antibodies; however, at their deepest, antigens did make much greater use of concavity formed by antibodies (i.e. had smaller concavity values), though more profoundly for Fab-bound antigens than for V_HH_-bound antigens (Supplementary Fig. S5 and Supplementary Table S6).

We further investigated whether antigen–antibody interface use of concavity was specific to certain amino acids (Supplementary Fig. S6 and Supplementary Table S7). While arginine was more present in antigens bound with more concave binding modes into especially Fab and scFv antibodies than other amino acids on average, all amino acid ANOVA groupings overlapped extensively, with the majority of amino acids grouped as having no statistically significantly difference by ANOVA. Average concavity for all residues was at the ‘flat’ end of the scale (>7.9 Å). However, with the exception of cysteine in Fab bound antigens and tryptophan and valine in scFv bound antigens, every amino acid sampled deep concavity space of <4.0 Å, indicating that there were instances of each amino acid in antigens that used antibody–formed concavities in the dataset (Supplementary Fig. S6 and Supplementary Table S7).

**Figure 5.**
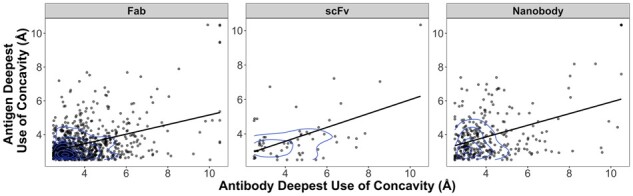
Point and 2D density distributions of deepest use of concavity by Fab, scFv, and V_HH_ antibodies and their antigens. Each point represents the deepest use of concavity on the antibody (abscissa) and antigen (ordinate) for an antibody–antigen complex.

**Figure 6.**
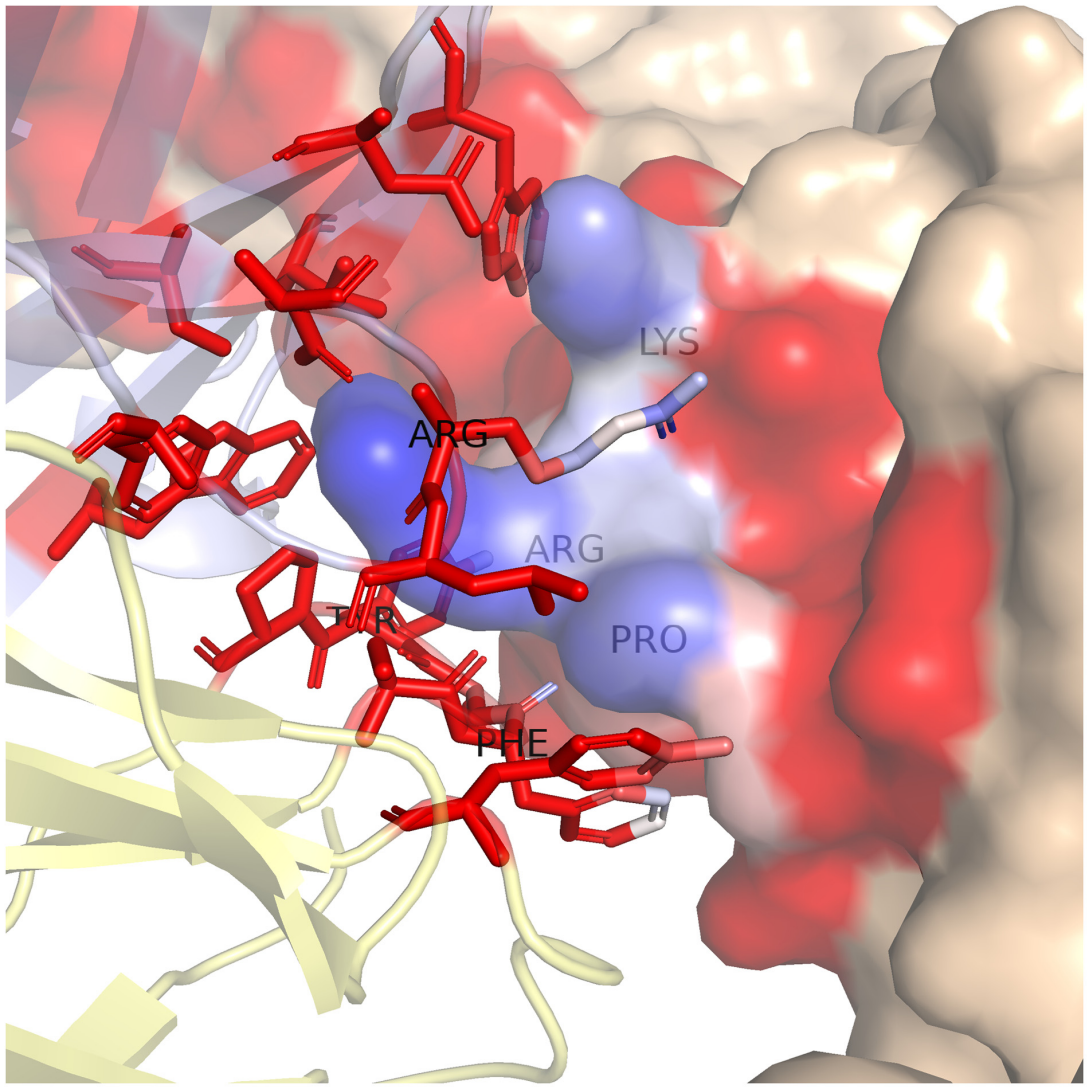
Interlocking deeply-bound residues in the macrophage colony-stimulating factor 1 receptor bound to an inhibitory Fab (PDB ID: 4LIQ). The antibody heavy chain is coloured in light blue, and the antibody light chain is coloured in light yellow. The macrophage colony-stimulating factor antigen is coloured in wheat. Antibody chains are shown in translucent cartoon representation, and part of the binding site of the antigen is shown in surface representation. The CDR residues within 5.0 Å of antigen interface are shown in stick representation. Both epitope and paratope residues are coloured by atomic concavity value on a rainbow scale, where red is no use of concavity (10.5 Å) and blue is high use of concavity (2.5 Å).

Given that the distributions of antibody CDR and antigen use of concavity at deepest (Supplementary Fig. S5) indicated that many antibody–antigen interfaces exploited concave binding modes on the antibody or antigen side of the interface, we tested whether extent of concavity was correlated between antibody sides of interfaces and antigen sides of interfaces. The antibody and antigen use of concavity were moderately correlated (Fig. 5) for scFv (*R* = 0.51, *P* = 1.0E–05) and V_HH_ (*R* = 0.40, *P* = 7.4E–14) and weakly correlated for Fab (*R* = 0.39, *P* < 2.2E–16). The 2D density distributions in Fig. 5 show that the densest area of the Fab and V_HH_ antibody–antigen complex point distribution lay within <3 Å concavity for both antibody and antigen use. For scFv antibody–antigen complexes, the 2D density distribution was more dispersed. A representative example is shown in Fig. 6 that arginine uses concave binding mode in both of Fab-H3 and antigen surface (Fig. 6).

### 3.7 Interatomic interactions in antibody CDRs

We further analysed the use of intermolecular interactions at antibody–antigen interfaces using Arpeggio. Supplementary Figure S7 shows how the use of interactions, normalized per 100 Å BSA, varied between antibody–antigen interfaces. ANOVA revealed that the prevalence of each individual interaction type was not statistically significantly different between antibody–antigen interfaces (Supplementary Fig. S7 and Supplementary Table S8). In all types of antibody–antigen interfaces, hydrophobic interactions were significantly more prevalent than polar/hydrogen bonding interactions (however, hydrophobic interactions have a measuring bias). Ring interactions exhibited no statistically significant difference in prevalence from one another, however all ring interactions were significantly less prevalent than polar/hydrogen bonding interactions (Supplementary Table S8).

## 4 Discussion

In this study, we have investigated the use of concavity and interatomic interactions to better understand antibody recognition at promiscuous antigen-antibody interfaces across Fab-, scFv-, and nanobody (V_HH_)- antigen complexes. All H3 CDRs showed distinctly longer CDRs with their use of deeper concavity, both on average and at deepest, than others (Supplementary Tables S1–S3). While V_HH_ CDR H3 showed the potential use of shallow pockets (Supplementary Fig. S8a and Supplementary Table S3), their average use of concavity was poor overall (>9 Å, Supplementary Table S2). Still, there was statistically significant difference in use of average concavity between V_H_V_L_ and V_HH_ CDR H3 regions (Supplementary Table S2), and especially V_HH_ CDR H3 regions make more use of concavity than Fab CDR H3 on average. To avoid underestimating the use of concavity, we checked deepest concavity of CDRs and all H3 CDRs showed the range of average of deepest concavity between 4.7 and 5.3 Å, which is significantly deeper than the 7.4–10.0 Å range of other CDRs (Supplementary Table S3). This may imply CDR regions were not extended into concavities in their entirety but tended to do so with only certain CDR residues.

The hypothesis that longer H3 CDR regions make more use of concavity than shorter CDR H3 regions is supported, albeit with only weak to moderate correlations (Fig. 3). The correlation of length and concavity usage was stronger for scFv antibody–antigen interfaces than for Fab or V_HH_ interfaces, implying promise for investigation of the use of single-chain antibodies and nanobodies in binding to concave binding sites, particularly in light of their apparent tendency towards longer CDR lengths. Though the correlations of L2, L3, and H2 of scFv were statistically significant (*P* < 0.05), they were not used in further analysis because they were associated with limited number of variables for CDR lengths (between one to three types of CDR lengths, Fig. 3).

While use of concavity based on antibody residue position (Supplementary Fig. S4) did not show statistically significant differences, two-chain antibodies, Fab and scFv, tend to use a wider range of CDR-H3 residues, 12–15 amino acids, for their concave binding modes than it of V_HH_, 4 amino acids. According to the analysis of amino acid concavity preferences, tryptophan in V_HH_-H3 showed (Fig. 4 and Supplementary Table S5) more use of concavity than any other residues. This further indicates no general answer to which residue positions or amino acids, and thus by proxy molecular shapes and physicochemistry, can be important in antibody paratopes utilizing concavity. The paucity of nonredundant data antibody–protein antigen interaction data in the PDB, in particular for scFv and V_HH_ antibodies, could be a factor to why potential trends, such as on average more use of concavity for phenylalanine, tryptophan, and tyrosine antibody residues, were not statistically significant.

Interestingly, Fab and V_HH_ antibodies showed both antibody and antigen deepest use of concavity is predominantly in the 2 to 4 Å range (Fig. 5), indicating that both sides of these interfaces engage in concavity with one another. The two-chain nature of V_H_V_L_ (Fab and scFv) enables the formation of interfacial pockets (Supplementary Fig. S8b), which may explain the use of deeper concavity on binding antibodies on average and at deepest by V_H_V_L_ antigens compared to V_HH_ antibodies (Supplementary Table S6). V_H_V_L_ engagement of concavity on both the antigen and antibody side may reflect an ‘interlocking anchor’ type phenomenon, where concavity on both sides of the interface are involved in protrusion of one or more residues into an anchor pocket for fast recognition as per the anchor hypothesis (Rajamani *et al.* 2004), such as in Fig. 6, however confirmation of this hypothesis would require further study.

The analysis of use of interatomic interactions in antibody–antigen complexes did not reveal significant differences in most cases between use of interactions by V_H_V_L_ and V_HH_ antibodies. This observation is especially interesting in that V_H_V_L_ antibodies include both heavy and light chains, and may be indicative of heavy chains performing the majority of interactions with the antigen epitope. The greater prevalence of ring–ring and ring–atom interactions from antibodies to antigens compared to atom-ring interactions may imply that antibody CDRs, which are typically tyrosine rich (Koide and Sidhu 2009, Ramaraj *et al.* 2012), utilize these functions in molecular recognition, and that they are likely important in antibody and/or small-molecule drug design against antigen epitopes; however the differences observed are not statistically significant and more observations may be necessary to draw firm conclusions.

Missing from this analysis is an understanding of protein and binding site dynamics. Further study of where antibodies and small-molecule ligands compete for the same binding site, or molecular dynamics studies of antigens’ binding sites, may reveal conformational differences including differing use of/opening of concavities. It would however be interesting to further study the landscapes of peptide and small-molecule interactions with antibodies, in particular with respect to antibody formation of concavity.

In conclusion, antibody–antigen interfaces tend to be flat on average but with dotted elements of use of concavity by single residues. The correlation between CDR length and use of deepest concavity suggests that V_HH_ may have an advantage in antigens with shallow pockets or grooves, but further study is required to determine whether it was affected by the limited number of V_HH_-antigen complexes. With respect to small-molecule drug design to modulate antigens at antigen epitope surfaces attempting to exploit multiple small-volume pockets may be prudent. For V_H_V_L_ antibody–antigen complexes, formation of concavity by the antibody that is utilized by the antigen is likely to be difficult to replicate with a small-molecule. The ‘interlocking’ phenomenon is not as apparent for V_HH_ antibody–antigen complexes compared to V_H_V_L_ antibodies, which may indicate that V_HH_ antibodies with long CDR H3 regions utilizing concavities, may be better frameworks for attempting conversion of antibody CDR regions to small-molecule drug binders. We believe that the concept of ‘use of concavity’ is not only useful for understanding the shape complementarity of the mutants and their target antigen surfaces in antibody design and engineering, but also for explaining the high structural plasticity of antibody–antigen interface landscapes.

## Supplementary Material

btad392_Supplementary_Data

## Data Availability

The data and scripts underlying this article are available at: https://github.com/YoochanMyung/scripts.
