# Supplementary material for: Understanding the complementarity and plasticity of antibody–antigen interfaces
Source: Bioinformatics. 2023 Jun 29;39(7):btad392. doi: 10.1093/bioinformatics/btad392 (PMC10329492; doi:10.1093/bioinformatics/btad392)
Supplement: btad392_Supplementary_Data [file btad392_supplementary_data.pdf]

SUPPORTING INFORMATION

Understanding the complementarity and plasticity of antibody-antigen interfaces

Yoochan Myung[1,2,3,5], Douglas E.V. Pires[2,3,4], David B. Ascher[1,2,3,5]

[1] Structural Biology and Bioinformatics, Department of Biochemistry and Pharmacology, University of Melbourne, Melbourne, VIC, Australia

[2] Computational Biology and Clinical Informatics, Baker Heart and Diabetes Institute, Melbourne, VIC, Australia

[3] Systems and Computational Biology, Bio21 Institute, University of Melbourne, Melbourne, VIC, Australia

[4] School of Computing and Information Systems, University of Melbourne, Melbourne, VIC, Australia

[5] School of Chemistry and Molecular Biosciences, University of Queensland, St Lucia, QLD, Australia

[*] To whom correspondence should be addressed D.B.A. Tel: +61 90354794; Email: d.ascher@uq.edu.au.

Correspondence may also be addressed to D.E.V.P. douglas.pires@unimelb.edu.au.

FIGURES

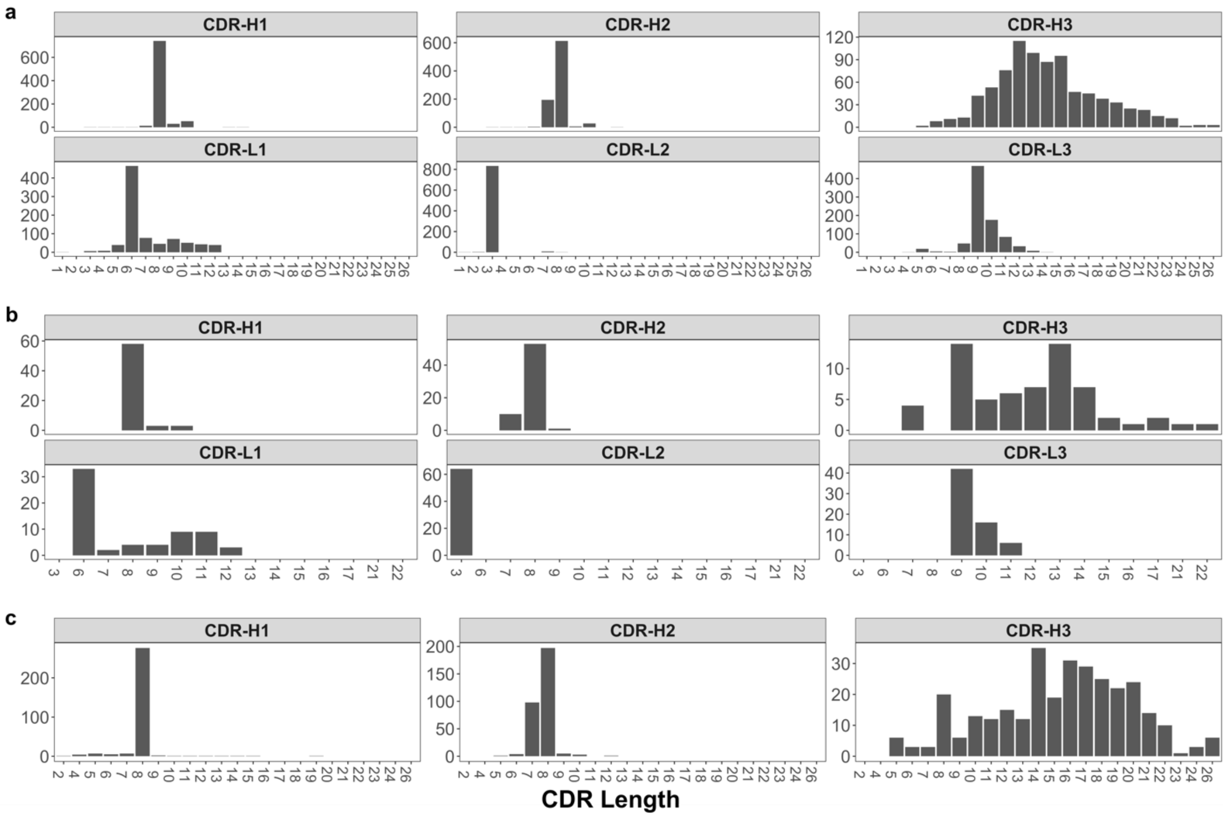

Figure S1. Histogram distributions of CDR lengths for antibodies. Histogram distributions of CDR lengths in Fab (a), scFv (b) and V_{HH} (c) antibodies.

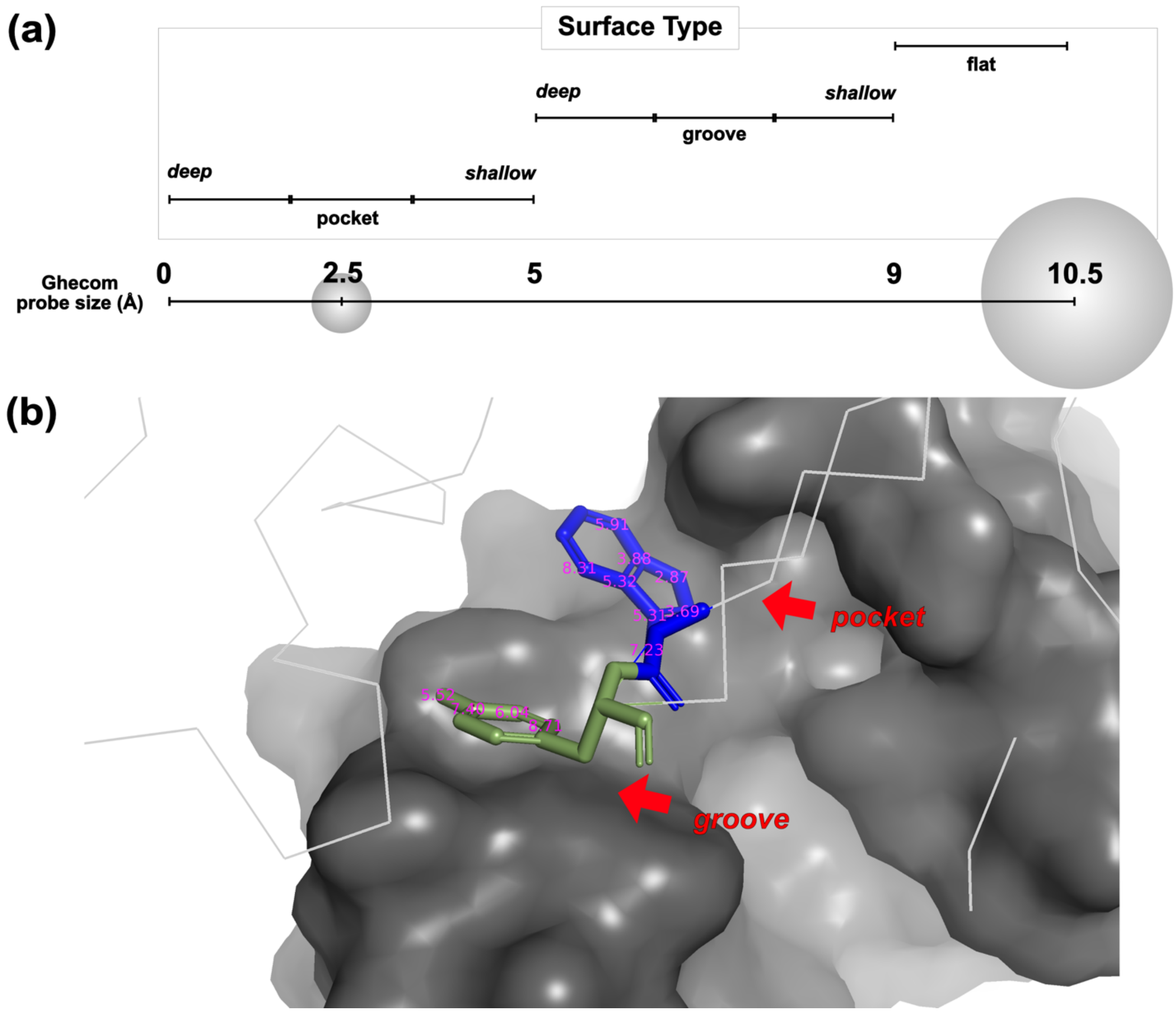

Figure S2. Types of protein binding surface based on Ghecom concavity measurement. (a) Ghecom measures the concavity of the protein surface using the different sizes of probes where smaller probes represent deeper protrusion into the binding surface and vice versa. (b) The two residues of antibody-antigen interface (PDB ID: 1DZB) are shown in coloured-stick representation with their Rinaccess values labelled in magenta. The Rinaccess of 2.87 of the tryptophan (blue stick) reflects the pocket surface of its binding surface, whereas the tyrosine (green stick) with Rinaccess values between 5.5 and 8.71 represent the shallow groove of the partner surface.

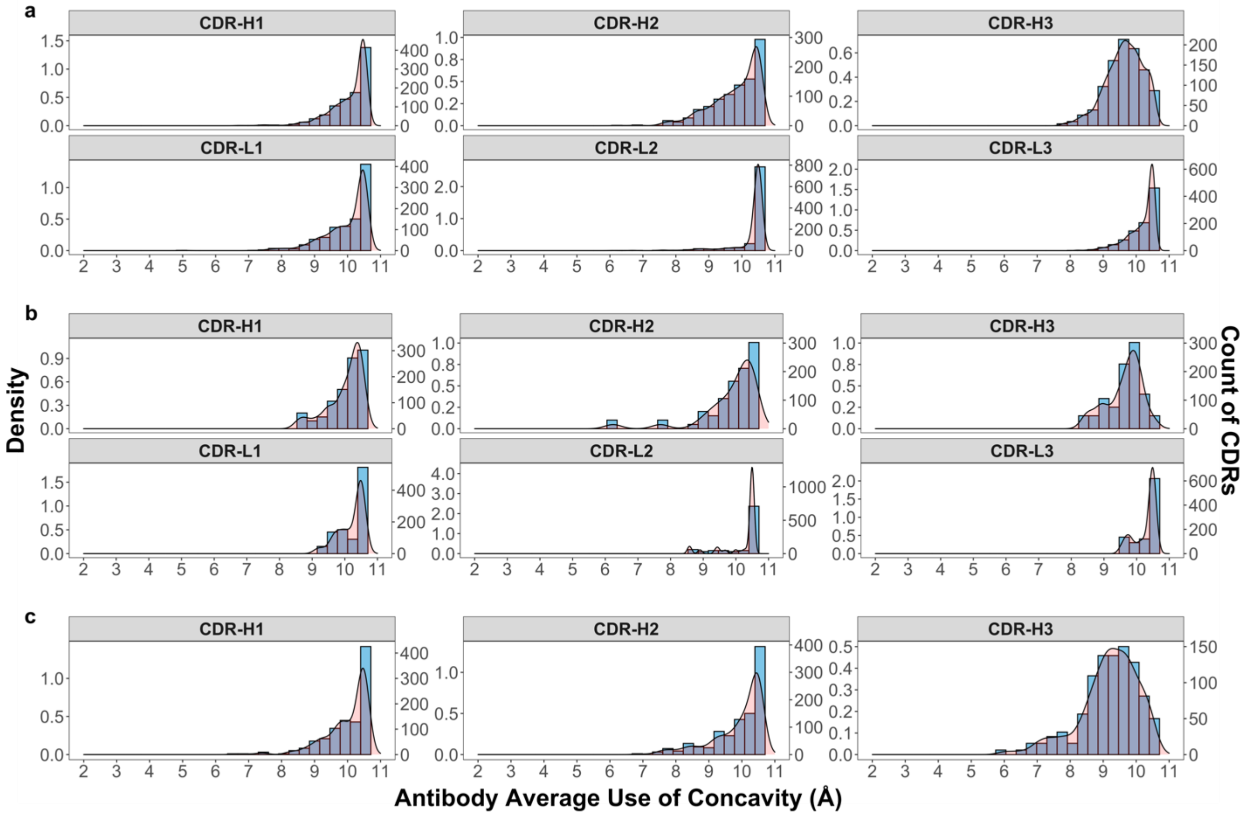

Figure S3. Histogram distributions of average use of concavityof CDR regions from antibodies in the SAbDab. Average concavity distributions for CDRs in Fab (a), scFv (b) and V_{HH}(c) antibodies. Concavity is as measured by Ghecom, representing the smallest spherical probe size that was able to enter a space around the partner protein's surface (where smaller values represent deeper binding). Concavity was assigned to CDR atoms and the smallest value (deepest protrusion into antigen) per-residue was measured for each CDR residue and averaged. Plots are faceted by CDR designation. The Density (left Y-axis) and Count of CDRs (right Y-axis) are shown in the red shaded curve and blue shaded bar, respectively.

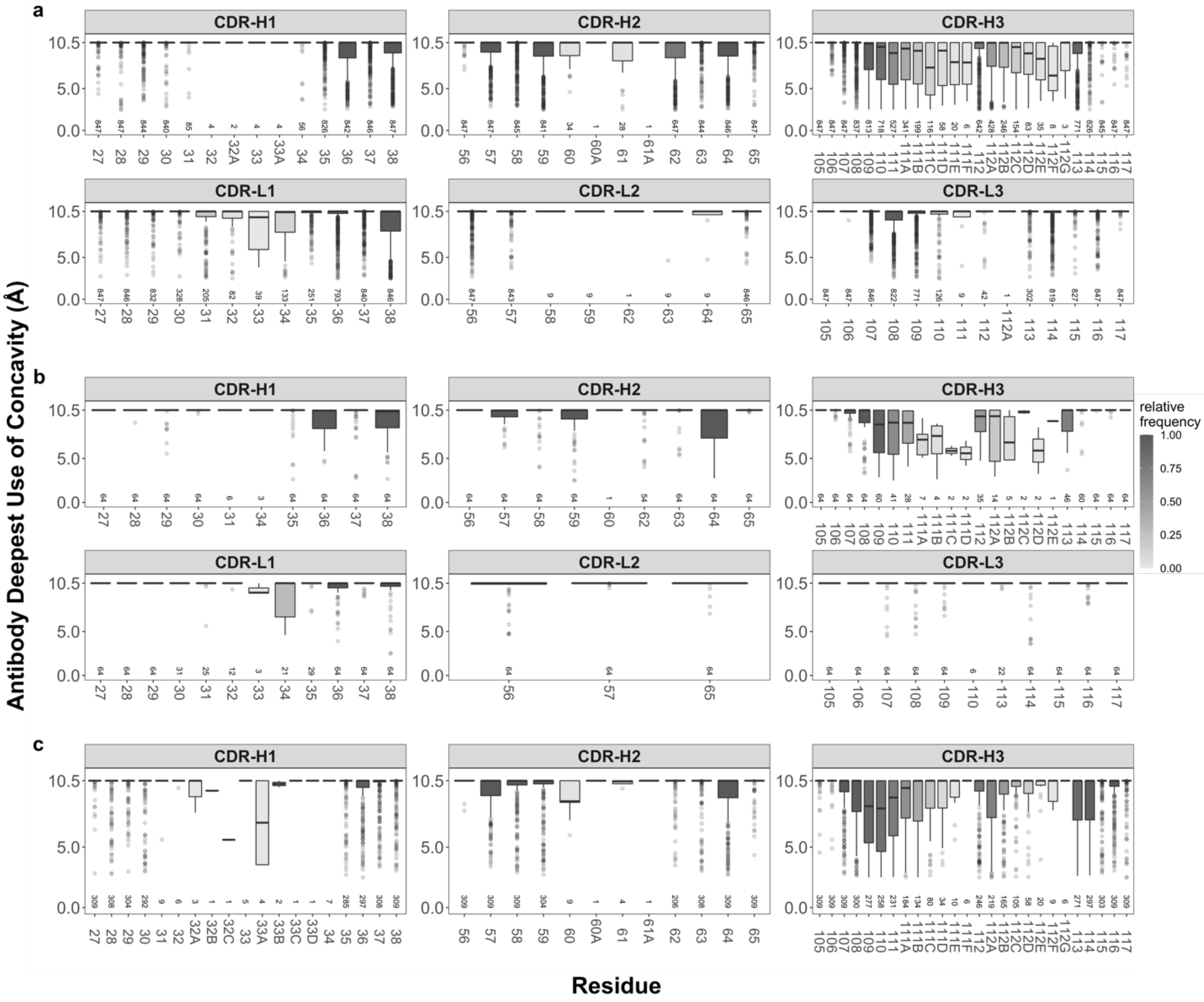

Figure S4. Boxplot distributions of residue use of concavity by position in CDRs Fab (a), scFv (b) and V_{HH} (c). The abscissa shows the IMGT annotation number for each CDR, with the number of observations denoted by a grey number above. Each boxplot is colored by the relative frequency of the residue position in the corresponding CDR region.

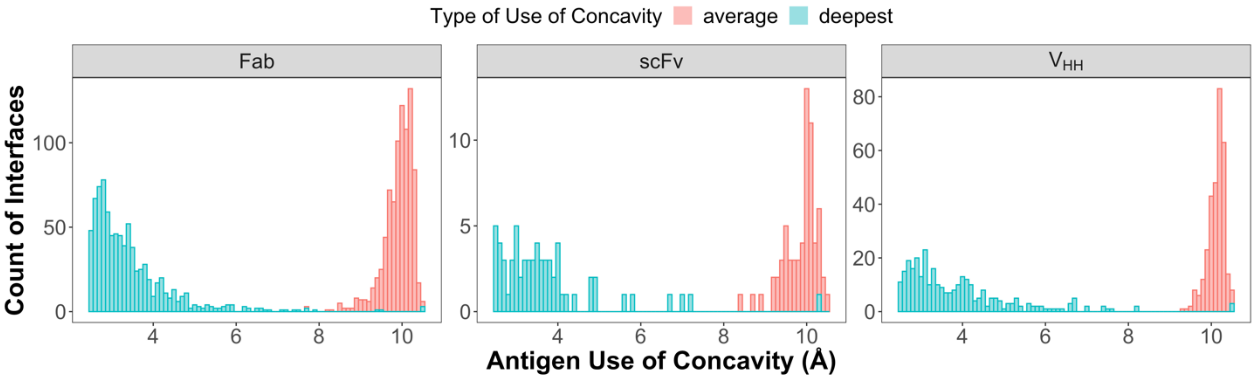

Figure S5. Histogram distributions of antigen use of concavity binding into Fab, scFv and V_{HH} antibodies. Distributions are shown for average (red) and deepest (blue) use of antibody concativities by antigens.

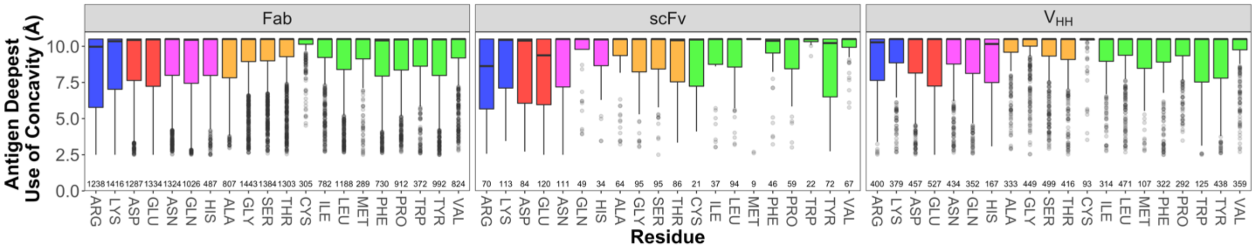

Figure S6. Boxplot distributions of antigen residue use of concavity by amino acid in Fab, scFv and V_{HH}. The use of deepest concavity of amino acids in epitopes across Fab, scFv and V_{HH} antibody-antigen complexes were measured. Residue distributions are coloured using a modified version of the Lesk colour scheme (positively charged = blue, negatively charged = red, polar = magenta, small non-polar = orange, hydrophobic = green).

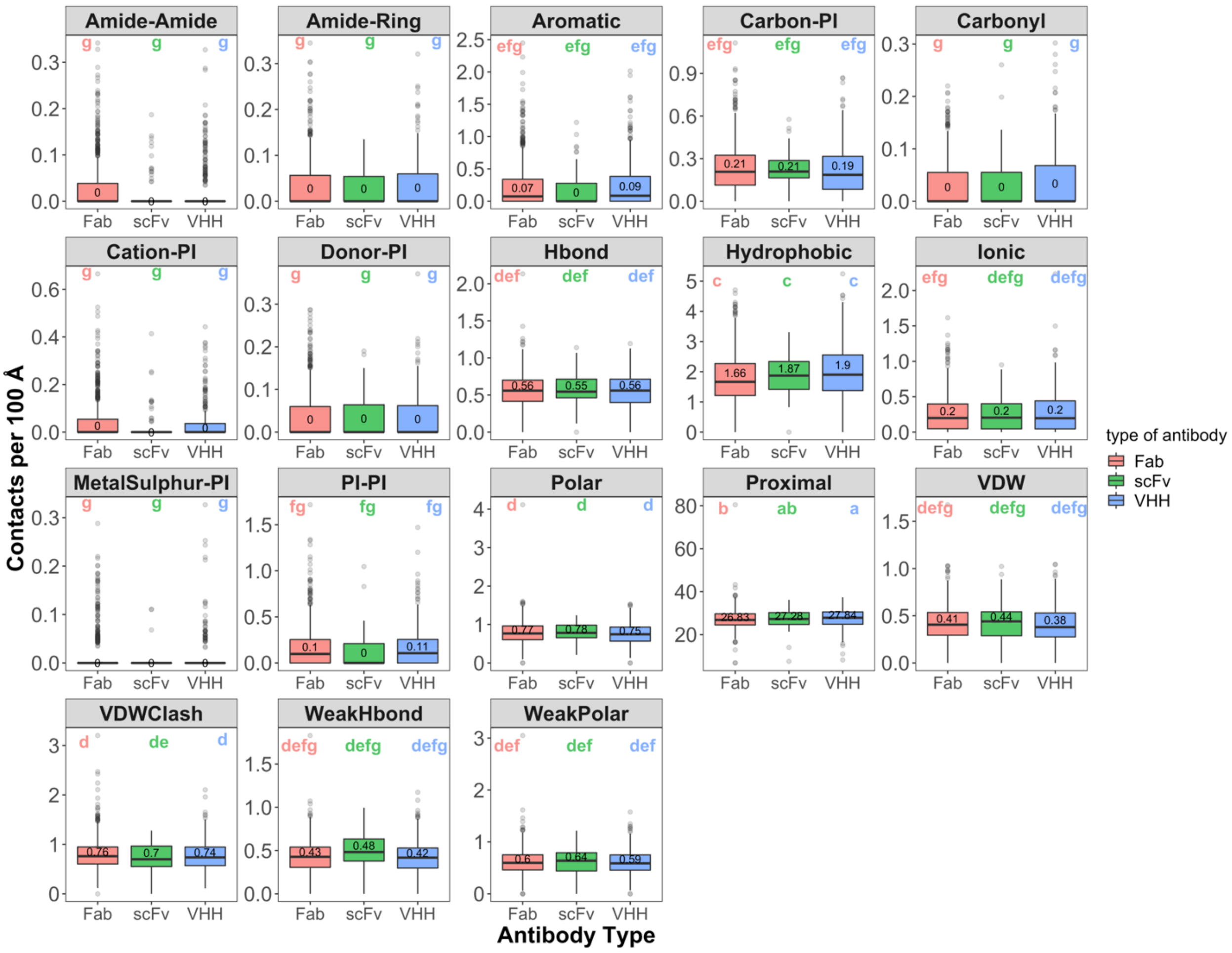

Figure S7. Boxplot distributions of Arpeggio structural interactions analysis of interatomic interactions per 100 Å. Individual data points are shown as grey circles, and ANOVA statistically significantly similar groups are indicated above boxes. Smaller letters (where a < b < c < d) indicate group means are significantly higher than groups denoted by larger letters.

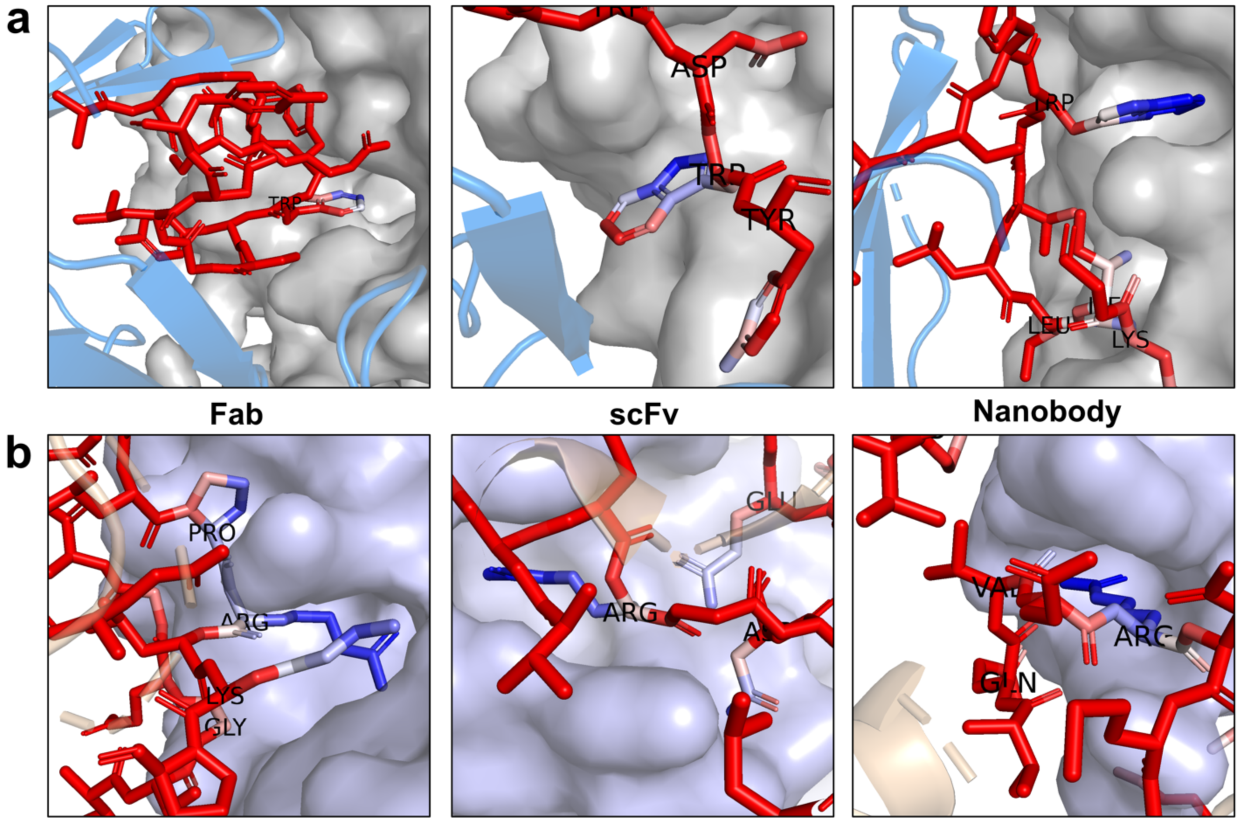

Figure S8. Structural examples of antibody use of concavity on binding into antigens and antigen use of concavity on binding into antibodies for Fab, scFv and V_{HH} antibody-antigen complexes. (a) Antigens are shown in surface representation and antibody chains are shown in translucent cartoon representation with light blue colour. CDR-H3 residues are shown in stick representation. On the contrary, (b) antibody chains shown in surface representation and antigen chains are shown in translucent cartoon representation with wheat colour. Antigen residues within 5.0 Å of antibody protomers are shown in stick representation. For both (a) and (b), the stick representation was coloured by atomic concavity value on a rainbow scale, where red is no use of concavity (10.5 Å) and blue is high use of concavity (2.5 Å).

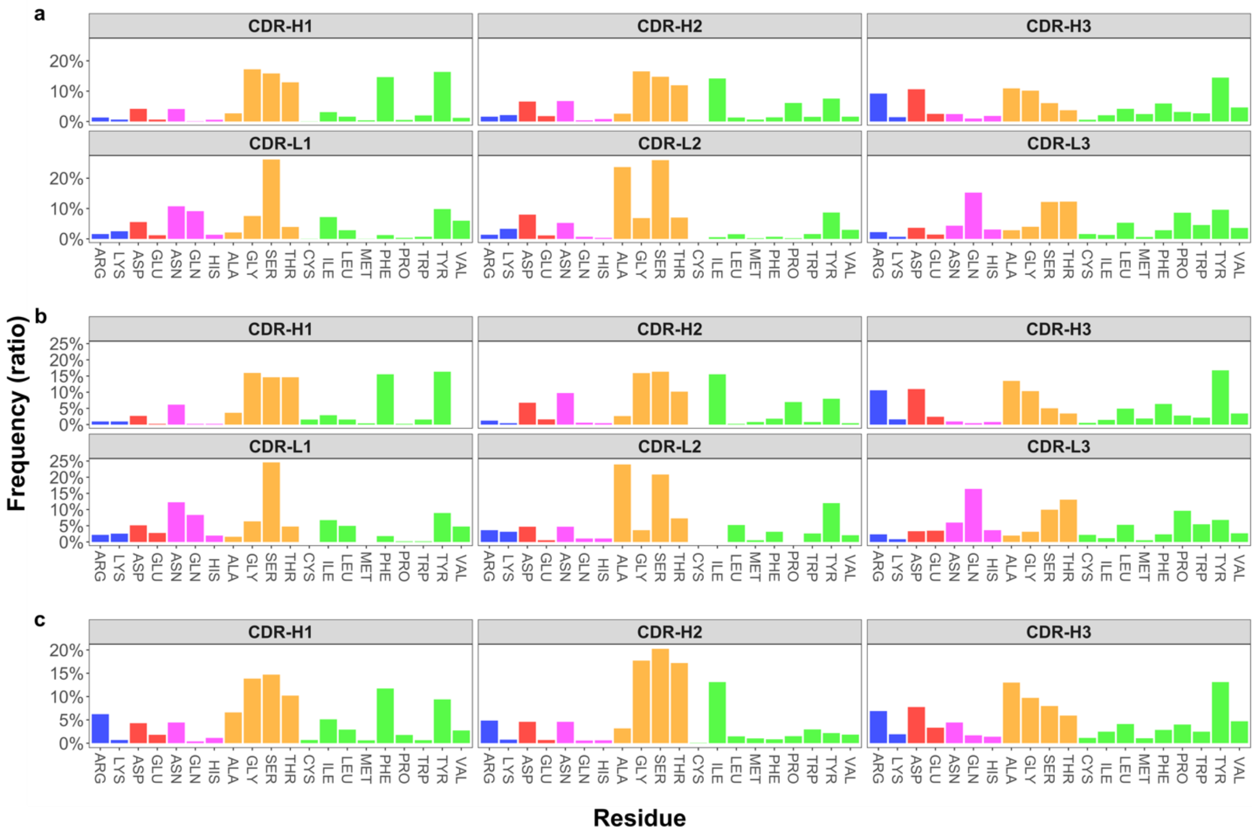

Figure S9. Histogram distributions of amino acid propensity in Fab (a), scFv (b) and V_{HH} (c). Residue distributions are coloured using a modified version of the Lesk colour scheme (positively charged = blue, negatively charged = red, polar = magenta, small non-polar = orange, hydrophobic = green).

TABLES

Table S1. Summary statistics and ANOVA analysis for antibody CDR length.

Ab Type	CDR	ANOVA Group	Mean	SD	n	Min	Med	Max	Mode	Range
V_{HH}	H3	a	15.4	4.51	309	5	16	26	14	21
Fab	H3	b	14.06	3.75	847	5	14	26	12	21
scFv	H3	c	11.83	2.97	64	7	12	22	9	15
scFv	L3	d	9.44	0.66	64	9	9	11	9	2
Fab	L3	d	9.39	1.22	847	4	9	14	9	10
Fab	H1	e	8.14	0.72	847	3	8	14	8	11
scFv	H1	e	8.14	0.47	64	8	8	10	8	2
V_{HH}	H1	e	7.94	1.21	309	2	8	19	8	17
scFv	L1	ef	7.89	2.19	64	6	6	12	6	6
scFv	H2	ef	7.86	0.39	64	7	8	9	8	2
Fab	H2	ef	7.83	0.65	847	3	8	12	8	9
V_{HH}	H2	ef	7.7	0.64	309	5	8	12	8	7
Fab	L1	f	7.13	1.96	847	1	6	12	6	11
Fab	L2	g	3.04	0.43	847	1	3	8	3	7
scFv	L2	g	3	0	64	3	3	3	3	0

Table S2. Summary statistics and ANOVA analysis for antibody average use of concavity (Å).

Ab Type	CDR	ANOVA Group	Mean	SD	n	Min	Med	Max	Mode	Range
Fab	L2	a	10.32	0.52	847	6.09	10.5	10.5	10.5	4.41
scFv	L3	ab	10.29	0.32	64	9.52	10.5	10.5	10.5	0.98
scFv	L1	abc	10.21	0.38	64	9.16	10.48	10.5	10.5	1.34
scFv	L2	abc	10.21	0.58	64	8.56	10.5	10.5	10.5	1.94
Fab	L3	bc	10.18	0.42	847	8.2	10.36	10.5	10.5	2.3
Fab	H1	bc	10.06	0.57	847	6.68	10.3	10.5	10.5	3.82
scFv	H1	bcd	10.01	0.54	64	8.56	10.23	10.5	10.5	1.94
V_{HH}	H1	cd	9.99	0.67	309	6.59	10.26	10.5	10.5	3.91
Fab	L1	cd	9.98	0.66	847	5.05	10.28	10.5	10.5	5.45
V_{HH}	H2	d	9.9	0.77	309	6.89	10.2	10.5	10.5	3.61
scFv	H2	de	9.85	0.9	64	6.23	10.14	10.5	10.5	4.27
Fab	H2	de	9.81	0.73	847	6.3	10.01	10.5	10.5	4.2
Fab	H3	e	9.64	0.55	847	7.81	9.68	10.5	10.5	2.69
scFv	H3	e	9.62	0.54	64	8.37	9.76	10.5	10.5	2.13
V_{HH}	H3	f	9.18	0.89	309	6	9.3	10.5	10.5	4.5

Table S3. Summary statistics and ANOVA analysis for antibody deepest use of concavity (Å).

Ab Type	CDR	ANOVA Group	Mean	SD	n	Min	Med	Max	Mode	Range
Fab	L2	a	9.99	1.36	847	2.73	10.5	10.5	10.5	7.77
scFv	L2	ab	9.66	1.67	64	4.69	10.5	10.5	10.5	5.81
scFv	L3	bc	8.9	2.33	64	3.61	10.5	10.5	10.5	6.89
scFv	L1	bc	8.64	2.38	64	2.5	10.37	10.5	10.5	8
Fab	L3	c	8.49	2.27	847	2.5	9.4	10.5	10.5	8
Fab	H1	c	8.13	2.51	847	2.5	9.07	10.5	10.5	8
Fab	L1	c	8.06	2.62	847	2.5	9.15	10.5	10.5	8
V_{HH}	H1	c	8.01	2.63	309	2.5	8.98	10.5	10.5	8
V_{HH}	H2	c	7.92	2.64	309	2.73	8.61	10.5	10.5	7.77
scFv	H1	cd	7.81	2.47	64	2.64	8.42	10.5	10.5	7.86
scFv	H2	cd	7.71	2.49	64	2.5	7.85	10.5	10.5	8
Fab	H2	d	7.39	2.67	847	2.5	7.69	10.5	10.5	8
scFv	H3	e	5.32	2.18	64	2.5	5.03	10.5	10.5	8
Fab	H3	e	5.23	2.35	847	2.5	4.46	10.5	10.5	8
V_{HH}	H3	e	4.66	2.09	309	2.5	3.92	10.5	10.5	8

Table S4. Summary statistics and ANOVA analysis for antibody residue position.

Ab Type	CDR	ANOVA Group	Residue Position	Mean	SD	n	Min	Med	Max	Mode	Range
Fab	H1	a	32	10.5	0	4	10.5	10.5	10.5	10.5	0
Fab	H1	a	32A	10.5	0	2	10.5	10.5	10.5	10.5	0
Fab	H1	a	33	10.5	0	4	10.5	10.5	10.5	10.5	0
Fab	H1	a	33A	10.5	0	4	10.5	10.5	10.5	10.5	0
V_{HH}	H1	a	33	10.5	0	5	10.5	10.5	10.5	10.5	0
V_{HH}	H1	ab	33C	10.5	NA	1	10.5	10.5	10.5	10.5	0
V_{HH}	H1	ab	33D	10.5	NA	1	10.5	10.5	10.5	10.5	0
V_{HH}	H1	ab	34	10.5	0	7	10.5	10.5	10.5	10.5	0
scFv	H1	ab	27	10.5	0	64	10.5	10.5	10.5	10.5	0
scFv	H1	ab	31	10.5	0	6	10.5	10.5	10.5	10.5	0
scFv	H1	ab	34	10.5	0	3	10.5	10.5	10.5	10.5	0
Fab	H2	ab	60A	10.5	NA	1	10.5	10.5	10.5	10.5	0
Fab	H2	ab	61A	10.5	NA	1	10.5	10.5	10.5	10.5	0
V_{HH}	H2	ab	60A	10.5	NA	1	10.5	10.5	10.5	10.5	0
V_{HH}	H2	ab	61A	10.5	NA	1	10.5	10.5	10.5	10.5	0
scFv	H2	ab	56	10.5	0	64	10.5	10.5	10.5	10.5	0
scFv	H2	ab	60	10.5	NA	1	10.5	10.5	10.5	10.5	0
Fab	H3	ab	105	10.5	0	847	10.5	10.5	10.5	10.5	0
V_{HH}	H3	ab	111F	10.5	0	6	10.5	10.5	10.5	10.5	0
V_{HH}	H3	ab	112G	10.5	0	6	10.5	10.5	10.5	10.5	0
scFv	H3	ab	105	10.5	0	64	10.5	10.5	10.5	10.5	0
scFv	H3	ab	115	10.5	0.01	64	10.4	10.5	10.5	10.5	0.1
scFv	H3	ab	117	10.5	0	64	10.5	10.5	10.5	10.5	0
scFv	L1	ab	27	10.5	0	64	10.5	10.5	10.5	10.5	0
scFv	L1	ab	28	10.5	0	64	10.5	10.5	10.5	10.5	0
scFv	L1	ab	29	10.5	0	64	10.5	10.5	10.5	10.5	0

scFv	L1	ab	30	10.5	0	31	10.5	10.5	10.5	10.5	0
Fab	L2	ab	58	10.5	0	9	10.5	10.5	10.5	10.5	0
Fab	L2	ab	59	10.5	0	9	10.5	10.5	10.5	10.5	0
Fab	L2	ab	62	10.5	NA	1	10.5	10.5	10.5	10.5	0
Fab	L3	ab	105	10.5	0	847	10.5	10.5	10.5	10.5	0
Fab	L3	ab	106	10.5	0.04	847	9.42	10.5	10.5	10.5	1.08
Fab	L3	ab	112A	10.5	NA	1	10.5	10.5	10.5	10.5	0
scFv	L3	ab	105	10.5	0	64	10.5	10.5	10.5	10.5	0
scFv	L3	ab	106	10.5	0	64	10.5	10.5	10.5	10.5	0
scFv	L3	ab	110	10.5	0	6	10.5	10.5	10.5	10.5	0
scFv	L3	ab	115	10.5	0	64	10.5	10.5	10.5	10.5	0
scFv	L3	ab	117	10.5	0	64	10.5	10.5	10.5	10.5	0
scFv	H1	ab	30	10.49	0.05	64	10.11	10.5	10.5	10.5	0.39
V_{HH}	H2	ab	56	10.49	0.18	309	8.01	10.5	10.5	10.5	2.49
scFv	H2	ab	65	10.49	0.04	64	10.24	10.5	10.5	10.5	0.26
scFv	L2	ab	57	10.49	0.08	64	9.94	10.5	10.5	10.5	0.56
Fab	L3	ab	117	10.49	0.1	847	8.36	10.5	10.5	10.5	2.14
scFv	H1	ab	28	10.48	0.18	64	9.08	10.5	10.5	10.5	1.42
Fab	H2	ab	56	10.48	0.19	847	7.22	10.5	10.5	10.5	3.28
scFv	H3	ab	116	10.48	0.11	64	9.67	10.5	10.5	10.5	0.83
Fab	H3	ab	116	10.47	0.28	847	5.49	10.5	10.5	10.5	5.01
Fab	H3	ab	115	10.46	0.41	845	3.33	10.5	10.5	10.5	7.17
Fab	H3	ab	117	10.46	0.34	847	5.34	10.5	10.5	10.5	5.16
Fab	L3	ab	115	10.45	0.47	827	3.09	10.5	10.5	10.5	7.41
Fab	H3	ab	106	10.44	0.38	847	6.64	10.5	10.5	10.5	3.86
V_{HH}	H3	ab	106	10.44	0.46	309	4.89	10.5	10.5	10.5	5.61
scFv	H3	ab	106	10.44	0.21	64	9.36	10.5	10.5	10.5	1.14
scFv	L1	ab	32	10.44	0.2	12	9.79	10.5	10.5	10.5	0.71
Fab	L2	ab	65	10.44	0.48	846	4.27	10.5	10.5	10.5	6.23

scFv	L3	ab	113	10.44	0.18	22	9.8	10.5	10.5	10.5	0.7
Fab	H1	ab	27	10.43	0.48	847	4.38	10.5	10.5	10.5	6.12
V_{HH}	H3	ab	105	10.43	0.48	309	4.55	10.5	10.5	10.5	5.95
Fab	H1	ab	30	10.41	0.67	840	2.88	10.5	10.5	10.5	7.62
V_{HH}	H1	ab	27	10.41	0.58	309	2.83	10.5	10.5	10.5	7.67
Fab	L1	ab	27	10.41	0.58	847	2.73	10.5	10.5	10.5	7.77
V_{HH}	H1	ab	32	10.4	0.24	6	9.9	10.5	10.5	10.5	0.6
scFv	L1	ab	37	10.4	0.34	64	8.94	10.5	10.5	10.5	1.56
Fab	L1	ab	29	10.39	0.68	832	2.92	10.5	10.5	10.5	7.58
Fab	L2	ab	57	10.39	0.66	843	3.7	10.5	10.5	10.5	6.8
Fab	H1	ab	28	10.38	0.81	847	2.5	10.5	10.5	10.5	8
scFv	L2	ab	65	10.37	0.57	64	7.03	10.5	10.5	10.5	3.47
Fab	L1	ab	28	10.36	0.71	846	2.9	10.5	10.5	10.5	7.6
V_{HH}	H2	ab	65	10.35	0.7	309	4.34	10.5	10.5	10.5	6.16
Fab	H2	ab	65	10.34	0.73	847	3.02	10.5	10.5	10.5	7.48
V_{HH}	H2	ab	61	10.31	0.31	4	9.85	10.44	10.5	10.5	0.65
Fab	L1	ab	30	10.31	0.85	328	2.74	10.5	10.5	10.5	7.76
scFv	H3	ab	112C	10.3	0.29	2	10.09	10.3	10.5	10.5	0.41
scFv	L3	ab	116	10.3	0.58	64	8.12	10.5	10.5	10.5	2.38
Fab	L3	ab	116	10.29	0.94	847	3.55	10.5	10.5	10.5	6.95
Fab	H1	ab	29	10.28	0.9	844	3.53	10.5	10.5	10.5	6.97
Fab	H1	ab	31	10.28	1.03	85	3.94	10.5	10.5	10.5	6.56
scFv	H3	ab	114	10.28	0.91	60	5.52	10.5	10.5	10.5	4.98
scFv	L1	ab	31	10.28	0.98	25	5.62	10.5	10.5	10.5	4.88
Fab	H3	ab	107	10.26	0.9	847	3.3	10.5	10.5	10.5	7.2
scFv	L1	ab	35	10.26	0.82	29	7.23	10.5	10.5	10.5	3.27
Fab	H3	ab	114	10.25	1.04	826	2.54	10.5	10.5	10.5	7.96
scFv	L3	ab	109	10.24	0.8	64	6.78	10.5	10.5	10.5	3.72
V_{HH}	H1	ab	33B	10.23	0.38	2	9.96	10.23	10.5	10.5	0.54

Fab	H2	ab	63	10.23	1.05	844	2.78	10.5	10.5	10.5	7.72
V_{HH}	H1	ab	29	10.22	0.97	304	2.97	10.5	10.5	10.5	7.53
scFv	H1	ab	29	10.21	0.91	64	5.56	10.5	10.5	10.5	4.94
Fab	H1	ab	35	10.2	1.02	826	2.52	10.5	10.5	10.5	7.98
Fab	L3	ab	107	10.16	0.95	846	4.55	10.5	10.5	10.5	5.95
scFv	H2	ab	63	10.13	1.21	64	5.38	10.5	10.5	10.5	5.12
V_{HH}	H2	ab	62	10.12	1.24	206	3.51	10.5	10.5	10.5	6.99
Fab	L2	ab	56	10.12	1.18	847	2.73	10.5	10.5	10.5	7.77
V_{HH}	H3	ab	117	10.11	1.31	309	2.5	10.5	10.5	10.5	8
scFv	L3	ab	107	10.11	1.28	64	4.44	10.5	10.5	10.5	6.06
Fab	L1	ab	37	10.1	1.16	840	2.81	10.5	10.5	10.5	7.69
Fab	L3	ab	113	10.1	1.23	302	2.68	10.5	10.5	10.5	7.82
Fab	H1	ab	37	10.05	1.26	846	2.85	10.5	10.5	10.5	7.65
Fab	H1	ab	34	10.03	1.54	56	3.69	10.5	10.5	10.5	6.81
V_{HH}	H1	ab	28	10.02	1.43	308	2.78	10.5	10.5	10.5	7.72
V_{HH}	H2	ab	63	10.02	1.51	308	3.03	10.5	10.5	10.5	7.47
Fab	L1	ab	35	10.02	1.13	251	4.31	10.5	10.5	10.5	6.19
V_{HH}	H1	ab	30	10	1.56	292	2.98	10.5	10.5	10.5	7.52
V_{HH}	H1	ab	35	10	1.49	285	2.72	10.5	10.5	10.5	7.78
scFv	L3	ab	108	9.99	1.38	64	4.71	10.5	10.5	10.5	5.79
scFv	H1	ab	35	9.98	1.54	64	2.64	10.5	10.5	10.5	7.86
V_{HH}	H1	ab	31	9.96	1.62	9	5.63	10.5	10.5	10.5	4.87
scFv	H3	ab	107	9.93	1.21	64	5.81	10.5	10.5	10.5	4.69
Fab	L3	ab	114	9.93	1.39	819	2.85	10.5	10.5	10.5	7.65
scFv	H2	ab	57	9.92	1.02	64	6.28	10.46	10.5	10.5	4.22
scFv	H2	ab	58	9.9	1.47	64	3.97	10.5	10.5	10.5	6.53
scFv	H1	ab	37	9.87	1.7	64	2.79	10.5	10.5	10.5	7.71
Fab	H3	ab	108	9.87	1.53	837	2.61	10.5	10.5	10.5	7.89
Fab	L3	ab	109	9.87	1.49	771	2.65	10.5	10.5	10.5	7.85

V_{HH}	H3	ab	112E	9.86	1.53	20	3.97	10.5	10.5	10.5	6.53
V_{HH}	H1	ab	38	9.85	1.57	309	3.08	10.5	10.5	10.5	7.42
Fab	H2	ab	58	9.85	1.61	845	2.91	10.5	10.5	10.5	7.59
V_{HH}	H3	ab	115	9.85	1.69	303	2.85	10.5	10.5	10.5	7.65
Fab	L2	ab	63	9.85	1.96	9	4.62	10.5	10.5	10.5	5.88
scFv	L3	ab	114	9.82	1.85	64	3.61	10.5	10.5	10.5	6.89
V_{HH}	H1	ab	37	9.81	1.59	308	3.38	10.5	10.5	10.5	7.12
Fab	L1	ab	36	9.81	1.58	793	2.5	10.5	10.5	10.5	8
scFv	H2	ab	62	9.78	1.74	54	4.01	10.5	10.5	10.5	6.49
scFv	L1	ab	33	9.77	0.63	3	9.4	9.42	10.5	9.42	1.1
scFv	L2	ab	56	9.76	1.63	64	4.69	10.5	10.5	10.5	5.81
V_{HH}	H3	ab	112C	9.74	1.71	105	2.57	10.5	10.5	10.5	7.93
scFv	L1	ab	36	9.72	1.51	64	3.91	10.5	10.5	10.5	6.59
V_{HH}	H2	ab	58	9.71	1.74	309	2.94	10.5	10.5	10.5	7.56
Fab	L2	ab	64	9.7	1.89	9	4.74	10.5	10.5	10.5	5.76
Fab	L3	ab	112	9.7	1.85	42	3.86	10.5	10.5	10.5	6.64
V_{HH}	H1	ab	32B	9.67	NA	1	9.67	9.67	9.67	9.67	0
V_{HH}	H3	ab	112F	9.66	1.08	9	8.05	10.5	10.5	10.5	2.45
V_{HH}	H3	ab	116	9.66	1.72	309	3.07	10.5	10.5	10.5	7.43
scFv	L1	ab	38	9.66	1.9	64	2.5	10.5	10.5	10.5	8
V_{HH}	H2	ab	59	9.65	1.87	304	2.74	10.5	10.5	10.5	7.76
Fab	H2	ab	57	9.64	1.55	847	2.88	10.5	10.5	10.5	7.62
V_{HH}	H1	ab	32A	9.62	1.52	3	7.86	10.5	10.5	10.5	2.64
V_{HH}	H2	ab	57	9.62	1.63	309	3.38	10.5	10.5	10.5	7.12
Fab	L3	ab	108	9.61	1.65	822	2.6	10.5	10.5	10.5	7.9
V_{HH}	H1	ab	36	9.59	1.84	297	2.5	10.5	10.5	10.5	8
Fab	H2	ab	60	9.59	1.6	34	4.59	10.5	10.5	10.5	5.91
V_{HH}	H3	ab	107	9.58	1.83	309	2.79	10.5	10.5	10.5	7.71
Fab	L3	ab	110	9.53	2.08	126	2.5	10.5	10.5	10.5	8

V_{HH}	H3	ab	112D	9.52	1.98	58	2.54	10.5	10.5	10.5	7.96
Fab	L3	ab	111	9.51	2.16	9	3.97	10.5	10.5	10.5	6.53
Fab	L1	ab	32	9.49	2.01	82	2.5	10.5	10.5	10.5	8
Fab	H3	ab	112	9.47	2.02	642	2.5	10.5	10.5	10.5	8
V_{HH}	H3	ab	111E	9.46	1.83	10	5.6	10.5	10.5	10.5	4.9
Fab	L1	ab	31	9.41	2.16	205	2.56	10.5	10.5	10.5	7.94
scFv	H1	ab	36	9.39	1.73	64	4.56	10.46	10.5	10.5	5.94
Fab	H1	ab	36	9.36	1.89	842	2.58	10.5	10.5	10.5	7.92
Fab	H1	ab	38	9.36	2.09	847	2.82	10.5	10.5	10.5	7.68
V_{HH}	H3	ab	112B	9.35	2.18	165	2.56	10.5	10.5	10.5	7.94
Fab	H2	ab	59	9.34	2.07	841	2.5	10.5	10.5	10.5	8
scFv	H2	ab	59	9.33	2.17	64	2.5	10.5	10.5	10.5	8
Fab	H3	ab	113	9.33	2.14	771	2.53	10.5	10.5	10.5	7.97
Fab	H2	ab	64	9.32	2.02	846	2.63	10.5	10.5	10.5	7.87
V_{HH}	H3	ab	112	9.32	2.27	246	2.5	10.5	10.5	10.5	8
V_{HH}	H2	ab	64	9.31	2.15	309	2.73	10.5	10.5	10.5	7.77
scFv	H3	ab	113	9.26	1.81	46	3.69	10.5	10.5	10.5	6.81
scFv	H3	ab	112E	9.25	NA	1	9.25	9.25	9.25	9.25	0
Fab	H2	ab	62	9.18	2.28	647	2.5	10.5	10.5	10.5	8
scFv	H3	ab	108	9.16	2.28	64	3.32	10.5	10.5	10.5	7.18
scFv	H3	ab	112	9.16	1.61	35	4.74	9.79	10.5	10.5	5.76
scFv	H1	ab	38	9.15	2	64	2.67	10.36	10.5	10.5	7.83
V_{HH}	H3	ab	111D	9.06	2.27	34	3.53	10.5	10.5	10.5	6.97
Fab	L1	ab	34	9.06	2.1	133	2.63	10.41	10.5	10.5	7.87
V_{HH}	H2	ab	60	9.05	1.64	9	5.98	8.8	10.5	10.5	4.52
V_{HH}	H3	ab	108	9.03	2.28	300	2.68	10.5	10.5	10.5	7.82
V_{HH}	H3	ab	111C	9.03	2.33	80	2.68	10.5	10.5	10.5	7.82
Fab	L1	ab	38	9.01	2.33	846	2.5	10.5	10.5	10.5	8
V_{HH}	H3	ab	112A	8.96	2.52	219	2.5	10.5	10.5	10.5	8

Fab	H2	ab	61	8.93	2.63	28	2.55	10.5	10.5	10.5	7.95
Fab	H3	ab	112A	8.93	2.32	428	2.56	10.48	10.5	10.5	7.94
V_{HH}	H3	ab	114	8.89	2.37	297	2.67	10.5	10.5	10.5	7.83
Fab	H3	ab	112B	8.86	2.36	246	2.67	10.48	10.5	10.5	7.83
V_{HH}	H3	ab	113	8.86	2.52	271	2.62	10.5	10.5	10.5	7.88
scFv	L1	ab	34	8.79	2.17	21	4.6	10.5	10.5	10.5	5.9
scFv	H2	ab	64	8.78	2.46	64	2.75	10.5	10.5	10.5	7.75
Fab	H3	ab	109	8.74	2.38	813	2.5	10.38	10.5	10.5	8
V_{HH}	H3	ab	111B	8.69	2.51	134	2.5	10.5	10.5	10.5	8
V_{HH}	H3	ab	111A	8.62	2.46	184	2.52	9.9	10.5	10.5	7.98
Fab	H3	ab	112C	8.51	2.5	154	2.5	9.96	10.5	10.5	8
scFv	H3	ab	111	8.35	2.2	28	4.08	9.06	10.5	10.5	6.42
Fab	H3	ab	112D	8.33	2.41	83	2.65	9.21	10.5	10.5	7.85
Fab	H3	ab	110	8.3	2.66	718	2.5	9.98	10.5	10.5	8
Fab	L1	ab	33	8.29	2.47	39	3.81	9.8	10.5	10.5	6.69
Fab	H3	ab	112G	8.27	3.86	3	3.81	10.5	10.5	10.5	6.69
Fab	H3	ab	111A	8.26	2.67	341	2.5	9.78	10.5	10.5	8
scFv	H3	ab	109	8.08	2.52	60	2.87	8.87	10.5	10.5	7.63
Fab	H3	ab	111B	8.07	2.7	199	2.68	9.52	10.5	10.5	7.82
Fab	H3	ab	111	8.02	2.78	527	2.5	9.25	10.5	10.5	8
Fab	H3	ab	112E	8.02	2.61	35	3.52	8.56	10.5	10.5	6.98
Fab	H3	ab	111D	8	2.85	58	2.98	9.54	10.5	10.5	7.52
V_{HH}	H3	ab	111	7.95	2.63	231	2.54	9.11	10.5	10.5	7.96
V_{HH}	H3	ab	109	7.78	2.72	277	2.5	8.39	10.5	10.5	8
Fab	H3	ab	111E	7.76	2.57	20	3.07	8.16	10.5	10.5	7.43
Fab	H3	ab	111F	7.7	3.17	6	3.47	8.12	10.5	10.5	7.03
scFv	H3	ab	110	7.68	3.12	41	2.5	9.08	10.5	10.5	8
scFv	H3	ab	112A	7.67	3.23	14	2.95	9.8	10.5	10.5	7.55
V_{HH}	H3	ab	110	7.5	2.85	258	2.5	8.2	10.5	10.5	8

scFv	H3	ab	112B	7.33	2.7	5	4.76	6.82	10.5	9.76	5.74
Fab	H3	ab	111C	7.26	2.98	116	2.5	7.52	10.5	10.5	8
Fab	H3	ab	112F	7.12	2.96	8	3.47	6.56	10.5	9.97	7.03
V_{HH}	H1	ab	33A	7.02	4.02	4	3.53	7.03	10.5	10.5	6.97
scFv	H3	ab	111A	6.88	1.66	7	5.1	7.14	9.59	9.59	4.49
scFv	H3	ab	111B	6.68	2.9	4	2.64	7.56	8.96	2.64	6.32
scFv	H3	ab	112D	5.89	3.73	2	3.26	5.89	8.53	8.53	5.27
scFv	H3	ab	111C	5.86	0.79	2	5.3	5.86	6.42	5.3	1.12
V_{HH}	H1	ab	32C	5.61	NA	1	5.61	5.61	5.61	5.61	0
scFv	H3	ab	111D	5.59	1.99	2	4.18	5.59	7	4.18	2.82

Table S5. Summary statistics and ANOVA analysis for antibody amino acid use of concavity in binding antigens.

Ab Type	CDR	ANOVA Group	Amino Acid	Mean	SD	n	Min	Med	Max	Mode	Range
Fab	H1	a	CYS	10.5	0	5	10.5	10.5	10.5	10.5	0
scFv	H1	a	CYS	10.5	0	8	10.5	10.5	10.5	10.5	0
scFv	H1	ab	GLU	10.5	NA	1	10.5	10.5	10.5	10.5	0
scFv	H1	ab	HIS	10.5	NA	1	10.5	10.5	10.5	10.5	0
scFv	H1	ab	LYS	10.5	0	5	10.5	10.5	10.5	10.5	0
scFv	H1	ab	PRO	10.5	NA	1	10.5	10.5	10.5	10.5	0
Fab	H2	ab	CYS	10.5	NA	1	10.5	10.5	10.5	10.5	0
V_{HH}	H2	ab	CYS	10.5	0	2	10.5	10.5	10.5	10.5	0
scFv	H2	ab	ARG	10.5	0	6	10.5	10.5	10.5	10.5	0
scFv	H2	ab	LEU	10.5	NA	1	10.5	10.5	10.5	10.5	0
scFv	H2	ab	LYS	10.5	0	2	10.5	10.5	10.5	10.5	0
scFv	H2	ab	MET	10.5	0	4	10.5	10.5	10.5	10.5	0
scFv	H2	ab	VAL	10.5	0	2	10.5	10.5	10.5	10.5	0
scFv	H3	ab	CYS	10.5	0	4	10.5	10.5	10.5	10.5	0
scFv	H3	ab	GLN	10.5	0	3	10.5	10.5	10.5	10.5	0
scFv	H3	ab	HIS	10.5	0	6	10.5	10.5	10.5	10.5	0
scFv	H3	ab	MET	10.5	0	14	10.5	10.5	10.5	10.5	0
Fab	L1	ab	CYS	10.5	0	2	10.5	10.5	10.5	10.5	0
scFv	L1	ab	ALA	10.5	0	8	10.5	10.5	10.5	10.5	0
scFv	L1	ab	GLN	10.5	0	42	10.5	10.5	10.5	10.5	0
scFv	L1	ab	GLU	10.5	0	14	10.5	10.5	10.5	10.5	0
scFv	L1	ab	ILE	10.5	0	34	10.5	10.5	10.5	10.5	0
scFv	L1	ab	LEU	10.5	0	25	10.5	10.5	10.5	10.5	0
scFv	L1	ab	PRO	10.5	NA	1	10.5	10.5	10.5	10.5	0
scFv	L1	ab	VAL	10.5	0	24	10.5	10.5	10.5	10.5	0
scFv	L2	ab	ALA	10.5	0.02	46	10.4	10.5	10.5	10.5	0.1

scFv	L2	ab	GLN	10.5	0	2	10.5	10.5	10.5	10.5	0
scFv	L2	ab	GLY	10.5	0	7	10.5	10.5	10.5	10.5	0
scFv	L2	ab	LEU	10.5	0	10	10.5	10.5	10.5	10.5	0
scFv	L2	ab	LYS	10.5	0	6	10.5	10.5	10.5	10.5	0
scFv	L2	ab	MET	10.5	NA	1	10.5	10.5	10.5	10.5	0
scFv	L2	ab	THR	10.5	0	14	10.5	10.5	10.5	10.5	0
scFv	L2	ab	VAL	10.5	0	4	10.5	10.5	10.5	10.5	0
Fab	L3	ab	CYS	10.5	0	127	10.5	10.5	10.5	10.5	0
scFv	L3	ab	ALA	10.5	0	12	10.5	10.5	10.5	10.5	0
scFv	L3	ab	ASN	10.5	0	36	10.5	10.5	10.5	10.5	0
scFv	L3	ab	CYS	10.5	0	13	10.5	10.5	10.5	10.5	0
scFv	L3	ab	GLN	10.5	0	99	10.5	10.5	10.5	10.5	0
scFv	L3	ab	MET	10.5	0	3	10.5	10.5	10.5	10.5	0
scFv	L3	ab	PRO	10.5	0	58	10.5	10.5	10.5	10.5	0
scFv	L3	ab	VAL	10.5	0	16	10.5	10.5	10.5	10.5	0
Fab	L2	ab	GLY	10.49	0.08	177	9.4	10.5	10.5	10.5	1.1
Fab	L2	ab	THR	10.49	0.05	180	9.86	10.5	10.5	10.5	0.64
Fab	L3	ab	GLN	10.49	0.2	1211	5.13	10.5	10.5	10.5	5.37
scFv	L1	ab	THR	10.48	0.1	24	10.02	10.5	10.5	10.5	0.48
Fab	L2	ab	ALA	10.48	0.18	609	6.96	10.5	10.5	10.5	3.54
Fab	L3	ab	PRO	10.48	0.15	684	7.81	10.5	10.5	10.5	2.69
scFv	L1	ab	GLY	10.47	0.12	32	9.94	10.5	10.5	10.5	0.56
scFv	L3	ab	HIS	10.47	0.12	22	9.92	10.5	10.5	10.5	0.58
Fab	L1	ab	VAL	10.46	0.47	363	3.34	10.5	10.5	10.5	7.16
Fab	L2	ab	PRO	10.46	0.11	6	10.24	10.5	10.5	10.5	0.26
scFv	L3	ab	ASP	10.46	0.17	20	9.74	10.5	10.5	10.5	0.76
scFv	L3	ab	SER	10.46	0.17	60	9.47	10.5	10.5	10.5	1.03
scFv	L1	ab	SER	10.45	0.28	124	8.63	10.5	10.5	10.5	1.87
scFv	H1	ab	LEU	10.44	0.14	8	10.11	10.5	10.5	10.5	0.39

Fab	L2	ab	SER	10.44	0.42	668	4.62	10.5	10.5	10.5	5.88
scFv	L2	ab	SER	10.44	0.26	40	8.97	10.5	10.5	10.5	1.53
scFv	H1	ab	PHE	10.43	0.38	81	8.14	10.5	10.5	10.5	2.36
scFv	H3	ab	LYS	10.43	0.22	12	9.75	10.5	10.5	10.5	0.75
scFv	H1	ab	GLN	10.42	NA	1	10.42	10.42	10.42	10.42	0
scFv	L1	ab	HIS	10.41	0.22	10	9.79	10.5	10.5	10.5	0.71
Fab	L2	ab	VAL	10.41	0.77	77	3.7	10.5	10.5	10.5	6.8
Fab	L1	ab	GLN	10.4	0.61	552	2.73	10.5	10.5	10.5	7.77
Fab	L3	ab	MET	10.4	0.55	48	6.79	10.5	10.5	10.5	3.71
Fab	L3	ab	VAL	10.4	0.51	284	4.98	10.5	10.5	10.5	5.52
Fab	H1	ab	GLY	10.39	0.57	1186	4.5	10.5	10.5	10.5	6
scFv	H1	ab	GLY	10.39	0.62	83	6.22	10.5	10.5	10.5	4.28
Fab	H2	ab	PRO	10.39	0.53	406	5.01	10.5	10.5	10.5	5.49
Fab	L1	ab	LEU	10.39	0.78	174	3.01	10.5	10.5	10.5	7.49
Fab	L3	ab	THR	10.39	0.6	977	3.28	10.5	10.5	10.5	7.22
scFv	L3	ab	LYS	10.39	0.25	5	9.93	10.5	10.5	10.5	0.57
V_{HH}	H1	ab	GLY	10.38	0.59	340	5.7	10.5	10.5	10.5	4.8
Fab	L1	ab	ILE	10.38	0.59	433	5.54	10.5	10.5	10.5	4.96
Fab	L2	ab	ASN	10.38	0.65	135	4.27	10.5	10.5	10.5	6.23
scFv	H2	ab	HIS	10.37	0	2	10.37	10.37	10.37	10.37	0
Fab	L3	ab	LEU	10.37	0.62	424	3.95	10.5	10.5	10.5	6.55
scFv	H3	ab	ALA	10.36	0.62	102	6.69	10.5	10.5	10.5	3.81
scFv	H2	ab	ALA	10.35	0.54	13	8.55	10.5	10.5	10.5	1.95
scFv	L1	ab	ASP	10.34	0.57	26	8.22	10.5	10.5	10.5	2.28
Fab	L2	ab	GLU	10.34	0.46	29	8.43	10.5	10.5	10.5	2.07
Fab	H1	ab	PHE	10.33	0.94	1008	2.86	10.5	10.5	10.5	7.64
Fab	L1	ab	GLU	10.32	0.78	73	5.78	10.5	10.5	10.5	4.72
scFv	L2	ab	ASN	10.32	0.37	9	9.45	10.5	10.5	10.5	1.05
V_{HH}	H2	ab	GLU	10.31	0.51	16	8.74	10.5	10.5	10.5	1.76

scFv	L1	ab	ASN	10.31	0.45	62	8.62	10.5	10.5	10.5	1.88
scFv	L3	ab	LEU	10.31	0.6	32	8.26	10.5	10.5	10.5	2.24
Fab	H1	ab	GLN	10.3	0.56	8	8.92	10.5	10.5	10.5	1.58
scFv	L2	ab	GLU	10.3	NA	1	10.3	10.3	10.3	10.3	0
Fab	L3	ab	ALA	10.3	0.83	228	3.86	10.5	10.5	10.5	6.64
scFv	H1	ab	ALA	10.29	0.51	19	8.41	10.5	10.5	10.5	2.09
V_{HH}	H2	ab	GLY	10.29	0.86	421	3.79	10.5	10.5	10.5	6.71
Fab	L3	ab	HIS	10.28	0.82	245	5.24	10.5	10.5	10.5	5.26
V_{HH}	H2	ab	PRO	10.27	0.67	36	7.85	10.5	10.5	10.5	2.65
scFv	L3	ab	THR	10.27	0.76	79	6.78	10.5	10.5	10.5	3.72
Fab	H1	ab	ALA	10.24	0.88	185	4.87	10.5	10.5	10.5	5.63
V_{HH}	H2	ab	ALA	10.24	1.03	76	3.85	10.5	10.5	10.5	6.65
V_{HH}	H2	ab	LYS	10.24	1.04	18	6.08	10.5	10.5	10.5	4.42
Fab	H2	ab	ILE	10.23	1.03	940	3.06	10.5	10.5	10.5	7.44
Fab	H2	ab	THR	10.23	0.99	793	3.02	10.5	10.5	10.5	7.48
Fab	H3	ab	ALA	10.23	1.12	1300	2.54	10.5	10.5	10.5	7.96
V_{HH}	H2	ab	ILE	10.22	1.18	312	3.03	10.5	10.5	10.5	7.47
scFv	L3	ab	PHE	10.22	0.71	14	8.33	10.5	10.5	10.5	2.17
Fab	H3	ab	LYS	10.21	1	167	3.46	10.5	10.5	10.5	7.04
Fab	L2	ab	ASP	10.21	1.01	206	3.65	10.5	10.5	10.5	6.85
V_{HH}	H1	ab	SER	10.2	1.08	361	2.72	10.5	10.5	10.5	7.78
scFv	H1	ab	TRP	10.2	0.4	8	9.43	10.36	10.5	10.49	1.07
Fab	H1	ab	THR	10.19	1.02	890	3.11	10.5	10.5	10.5	7.39
V_{HH}	H1	ab	THR	10.19	1.12	250	2.97	10.5	10.5	10.5	7.53
Fab	H2	ab	GLY	10.19	1.07	1094	2.88	10.5	10.5	10.5	7.62
Fab	L1	ab	ALA	10.19	1.1	129	2.87	10.5	10.5	10.5	7.63
Fab	L1	ab	SER	10.19	1.04	1582	3.08	10.5	10.5	10.5	7.42
Fab	L3	ab	LYS	10.19	1.01	55	4.71	10.5	10.5	10.5	5.79
Fab	H1	ab	VAL	10.17	1.03	82	5.82	10.5	10.5	10.5	4.68

V_{HH}	H1	ab	ALA	10.16	0.97	162	5.99	10.5	10.5	10.5	4.51
Fab	L1	ab	ASP	10.16	1.06	334	3.47	10.5	10.5	10.5	7.03
Fab	L3	ab	SER	10.16	1.07	968	3.27	10.5	10.5	10.5	7.23
scFv	H2	ab	ASP	10.15	1	34	5.32	10.5	10.5	10.5	5.18
Fab	L2	ab	ILE	10.15	0.76	14	8.37	10.5	10.5	10.5	2.13
scFv	L2	ab	TYR	10.15	1.15	23	5.8	10.5	10.5	10.5	4.7
V_{HH}	H2	ab	GLN	10.14	1.26	14	5.76	10.5	10.5	10.5	4.74
scFv	H2	ab	GLY	10.14	1.29	80	4.4	10.5	10.5	10.5	6.1
scFv	H2	ab	ILE	10.14	1.31	78	3.6	10.5	10.5	10.5	6.9
Fab	L1	ab	GLY	10.14	1.07	454	4.06	10.5	10.5	10.5	6.44
Fab	L3	ab	GLY	10.14	1	316	4.1	10.5	10.5	10.5	6.4
Fab	H1	ab	ILE	10.13	1.15	214	3.66	10.5	10.5	10.5	6.84
V_{HH}	H1	ab	PRO	10.13	0.99	43	6.44	10.5	10.5	10.5	4.06
Fab	L2	ab	LEU	10.12	1.13	40	5.43	10.5	10.5	10.5	5.07
Fab	H1	ab	LEU	10.1	1.29	111	3.39	10.5	10.5	10.5	7.11
Fab	H1	ab	SER	10.09	1.17	1091	2.58	10.5	10.5	10.5	7.92
V_{HH}	H1	ab	CYS	10.09	1.21	17	5.84	10.5	10.5	10.5	4.66
scFv	H2	ab	SER	10.09	0.94	82	5.97	10.5	10.5	10.5	4.53
scFv	L2	ab	ASP	10.09	0.51	9	9.16	10.47	10.5	10.5	1.34
Fab	L3	ab	ASP	10.09	1.25	292	3.39	10.5	10.5	10.5	7.11
Fab	L1	ab	LYS	10.08	1.27	152	2.5	10.5	10.5	10.5	8
Fab	L1	ab	MET	10.08	0.73	3	9.23	10.5	10.5	10.5	1.27
V_{HH}	H1	ab	ASP	10.07	1.26	105	4.79	10.5	10.5	10.5	5.71
scFv	H1	ab	THR	10.07	1.04	76	5.84	10.5	10.5	10.5	4.66
Fab	H2	ab	ALA	10.07	1.15	170	4.16	10.5	10.5	10.5	6.34
scFv	H1	ab	SER	10.06	1.29	76	4.29	10.5	10.5	10.5	6.21
Fab	H3	ab	MET	10.06	1.51	292	2.93	10.5	10.5	10.5	7.57
Fab	L1	ab	THR	10.06	1.28	237	3.05	10.5	10.5	10.5	7.45
V_{HH}	H1	ab	PHE	10.05	1.47	288	2.98	10.5	10.5	10.5	7.52

V_{HH}	H1	ab	VAL	10.05	1.15	67	5.05	10.5	10.5	10.5	5.45
scFv	H2	ab	PRO	10.05	1.55	35	3.97	10.5	10.5	10.5	6.53
Fab	L1	ab	ASN	10.05	1.23	648	2.74	10.5	10.5	10.5	7.76
Fab	L2	ab	GLN	10.05	1.09	17	7.14	10.5	10.5	10.5	3.36
Fab	L3	ab	ILE	10.05	1.32	104	3.2	10.5	10.5	10.5	7.3
Fab	H2	ab	LYS	10.04	1.13	139	5.18	10.5	10.5	10.5	5.32
V_{HH}	H2	ab	THR	10.04	1.29	409	3.15	10.5	10.5	10.5	7.35
scFv	H2	ab	THR	10.04	1.36	51	4.65	10.5	10.5	10.5	5.85
V_{HH}	H2	ab	MET	10.02	1.46	24	3.84	10.5	10.5	10.5	6.66
scFv	H3	ab	ASP	10.02	1.12	83	5.42	10.5	10.5	10.5	5.08
scFv	L3	ab	GLY	10	1.31	19	5.51	10.5	10.5	10.5	4.99
V_{HH}	H2	ab	SER	9.99	1.38	481	3.11	10.5	10.5	10.5	7.39
Fab	L3	ab	PHE	9.99	1.33	229	2.65	10.5	10.5	10.5	7.85
Fab	H3	ab	ASP	9.98	1.47	1266	2.5	10.5	10.5	10.5	8
V_{HH}	H3	ab	ALA	9.98	1.54	619	2.68	10.5	10.5	10.5	7.82
Fab	L2	ab	LYS	9.97	1.56	85	2.73	10.5	10.5	10.5	7.77
Fab	H1	ab	HIS	9.96	1.25	39	3.57	10.5	10.5	10.5	6.93
scFv	H2	ab	GLN	9.96	0.61	3	9.3	10.09	10.5	10.09	1.2
Fab	H1	ab	PRO	9.94	1.37	34	5.31	10.5	10.5	10.5	5.19
Fab	H1	ab	LYS	9.93	1.44	43	4.71	10.5	10.5	10.5	5.79
scFv	H3	ab	ARG	9.92	1.48	80	2.86	10.5	10.5	10.5	7.64
Fab	H1	ab	ASN	9.91	1.49	285	2.52	10.5	10.5	10.5	7.98
V_{HH}	H1	ab	GLU	9.91	1.59	44	3.08	10.5	10.5	10.5	7.42
V_{HH}	H1	ab	LEU	9.91	1.53	72	3.81	10.5	10.5	10.5	6.69
scFv	L1	ab	PHE	9.91	1.77	9	5.19	10.5	10.5	10.5	5.31
scFv	L3	ab	GLU	9.89	1.91	21	4.07	10.5	10.5	10.5	6.43
scFv	L3	ab	TRP	9.88	1.57	33	4.71	10.5	10.5	10.5	5.79
scFv	H1	ab	ILE	9.87	1.68	15	5.24	10.5	10.5	10.5	5.26
Fab	H1	ab	ASP	9.86	1.59	286	3.65	10.5	10.5	10.5	6.85

V_{HH}	H1	ab	ILE	9.85	1.55	125	3.47	10.5	10.5	10.5	7.03
scFv	L3	ab	TYR	9.85	1.57	41	3.61	10.5	10.5	10.5	6.89
Fab	H2	ab	SER	9.84	1.48	977	3.25	10.5	10.5	10.5	7.25
Fab	H3	ab	PHE	9.84	1.76	708	2.62	10.5	10.5	10.5	7.88
V_{HH}	H3	ab	CYS	9.84	1.44	54	4.94	10.5	10.5	10.5	5.56
scFv	H3	ab	PHE	9.84	1.83	48	2.55	10.5	10.5	10.5	7.95
V_{HH}	H2	ab	ASP	9.83	1.68	109	3.14	10.5	10.5	10.5	7.36
scFv	H3	ab	ILE	9.83	1.46	11	5.67	10.5	10.5	10.5	4.83
scFv	H2	ab	ASN	9.82	1.62	49	3.82	10.5	10.5	10.5	6.68
Fab	L2	ab	PHE	9.82	1.32	17	6.72	10.5	10.5	10.5	3.78
Fab	H2	ab	VAL	9.81	1.25	104	3.88	10.5	10.5	10.5	6.62
V_{HH}	H3	ab	LYS	9.81	1.67	91	2.62	10.5	10.5	10.5	7.88
V_{HH}	H1	ab	LYS	9.8	1.88	17	3.29	10.5	10.5	10.5	7.21
Fab	H3	ab	GLY	9.8	1.62	1208	2.5	10.5	10.5	10.5	8
Fab	L2	ab	TYR	9.79	1.6	223	3.2	10.5	10.5	10.5	7.3
Fab	L3	ab	ASN	9.79	1.55	346	2.73	10.5	10.5	10.5	7.77
Fab	H1	ab	MET	9.78	1.97	28	4.18	10.5	10.5	10.5	6.32
Fab	L1	ab	PRO	9.77	1.29	19	5.73	10.37	10.5	10.5	4.77
V_{HH}	H2	ab	VAL	9.76	1.69	44	3.9	10.5	10.5	10.5	6.6
Fab	L3	ab	ARG	9.72	1.72	178	2.81	10.5	10.5	10.5	7.69
Fab	H3	ab	ARG	9.71	1.9	1096	2.5	10.5	10.5	10.5	8
Fab	H2	ab	MET	9.7	1.66	42	4.3	10.5	10.5	10.5	6.2
Fab	L3	ab	TYR	9.7	1.69	763	2.5	10.5	10.5	10.5	8
Fab	H3	ab	THR	9.68	1.77	444	3.01	10.5	10.5	10.5	7.49
scFv	H1	ab	TYR	9.67	1.9	85	2.67	10.5	10.5	10.5	7.83
Fab	H3	ab	VAL	9.67	1.88	554	2.6	10.5	10.5	10.5	7.9
Fab	H1	ab	TYR	9.65	1.9	1126	2.5	10.5	10.5	10.5	8
V_{HH}	H1	ab	GLN	9.65	2.06	10	4.09	10.5	10.5	10.5	6.41
V_{HH}	H1	ab	TYR	9.65	1.85	230	3.38	10.5	10.5	10.5	7.12

V_{HH}	H3	ab	ASP	9.64	1.91	369	2.75	10.5	10.5	10.5	7.75
Fab	L2	ab	TRP	9.64	1.67	41	3.48	10.5	10.5	10.5	7.02
V_{HH}	H3	ab	ASN	9.61	1.91	211	2.5	10.5	10.5	10.5	8
scFv	H3	ab	PRO	9.61	2.09	21	4.18	10.5	10.5	10.5	6.32
Fab	L3	ab	TRP	9.61	1.66	363	2.68	10.5	10.5	10.5	7.82
Fab	H3	ab	SER	9.6	1.89	723	2.78	10.5	10.5	10.5	7.72
Fab	H3	ab	PRO	9.58	1.93	372	2.84	10.5	10.5	10.5	7.66
V_{HH}	H3	ab	SER	9.57	1.91	380	2.82	10.5	10.5	10.5	7.68
Fab	H2	ab	ASN	9.56	1.74	446	3.57	10.5	10.5	10.5	6.93
Fab	H2	ab	ASP	9.56	1.86	435	2.63	10.5	10.5	10.5	7.87
V_{HH}	H3	ab	HIS	9.54	1.87	66	2.76	10.5	10.5	10.5	7.74
Fab	H3	ab	HIS	9.52	1.93	216	2.56	10.5	10.5	10.5	7.94
Fab	L2	ab	ARG	9.52	1.94	35	4.88	10.5	10.5	10.5	5.62
scFv	H3	ab	VAL	9.5	2.19	26	2.95	10.5	10.5	10.5	7.55
Fab	L3	ab	GLU	9.48	2	111	3.55	10.5	10.5	10.5	6.95
Fab	H3	ab	CYS	9.46	1.66	68	5.29	10.5	10.5	10.5	5.21
V_{HH}	H2	ab	ASN	9.45	1.84	109	3.45	10.5	10.5	10.5	7.05
Fab	H3	ab	GLN	9.45	2.06	117	2.76	10.5	10.5	10.5	7.74
V_{HH}	H1	ab	ARG	9.44	2.19	153	2.78	10.5	10.5	10.5	7.72
V_{HH}	H1	ab	HIS	9.43	1.92	28	4.28	10.5	10.5	10.5	6.22
scFv	H3	ab	LEU	9.42	1.92	37	4.48	10.46	10.5	10.5	6.02
scFv	H1	ab	MET	9.41	0.23	2	9.24	9.41	9.57	9.24	0.33
Fab	H3	ab	ASN	9.41	2.21	289	2.51	10.5	10.5	10.5	7.99
Fab	L1	ab	ARG	9.4	1.96	96	2.5	10.5	10.5	10.5	8
Fab	H3	ab	GLU	9.38	2.12	301	2.75	10.5	10.5	10.5	7.75
scFv	H3	ab	GLY	9.38	1.94	78	3.93	10.5	10.5	10.5	6.57
V_{HH}	H1	ab	ASN	9.37	2.05	109	3.16	10.5	10.5	10.5	7.34
Fab	H2	ab	GLU	9.37	2.24	116	2.96	10.5	10.5	10.5	7.54
V_{HH}	H3	ab	THR	9.36	2.04	282	3.27	10.5	10.5	10.5	7.23

Fab	L1	ab	HIS	9.35	1.85	81	2.81	10.5	10.5	10.5	7.69
V_{HH}	H3	ab	GLY	9.31	2.02	462	2.85	10.5	10.5	10.5	7.65
scFv	H3	ab	ASN	9.29	2.32	7	4.43	10.5	10.5	10.5	6.07
Fab	H3	ab	LEU	9.28	2.16	497	2.68	10.5	10.5	10.5	7.82
scFv	L3	ab	ARG	9.27	2.13	14	4.44	10.41	10.5	10.5	6.06
Fab	H2	ab	HIS	9.26	2.15	53	2.89	10.5	10.5	10.5	7.61
V_{HH}	H2	ab	LEU	9.25	2.22	35	3.92	10.5	10.5	10.5	6.58
V_{HH}	H3	ab	PRO	9.24	2.04	190	3.22	10.5	10.5	10.5	7.28
scFv	H3	ab	SER	9.23	2.16	38	2.64	10.5	10.5	10.5	7.86
V_{HH}	H2	ab	ARG	9.2	2.28	115	2.73	10.5	10.5	10.5	7.77
V_{HH}	H3	ab	GLN	9.2	2.41	81	2.7	10.5	10.5	10.5	7.8
scFv	H1	ab	ASN	9.14	1.99	32	4.54	10.5	10.5	10.5	5.96
scFv	H2	ab	PHE	9.14	1.65	9	6.91	10.5	10.5	10.5	3.59
Fab	H1	ab	GLU	9.13	2.36	43	3.79	10.5	10.5	10.5	6.71
Fab	L1	ab	PHE	9.11	2.21	76	2.76	10.5	10.5	10.5	7.74
Fab	H3	ab	ILE	9.09	2.33	245	2.9	10.5	10.5	10.5	7.6
Fab	H2	ab	LEU	9.08	2.18	87	3.04	10.5	10.5	10.5	7.46
V_{HH}	H3	ab	GLU	9.06	2.24	158	2.78	10.49	10.5	10.5	7.72
Fab	L2	ab	HIS	9.04	2.82	8	3.11	10.5	10.5	10.5	7.39
V_{HH}	H3	ab	VAL	9.03	2.31	224	2.8	10.5	10.5	10.5	7.7
V_{HH}	H1	ab	MET	9.01	2.3	15	3.92	10.5	10.5	10.5	6.58
Fab	H1	ab	ARG	9	2.37	91	2.95	10.5	10.5	10.5	7.55
scFv	L1	ab	LYS	9	1.85	13	5.32	10.4	10.5	10.5	5.18
V_{HH}	H3	ab	TYR	8.99	2.46	624	2.5	10.5	10.5	10.5	8
V_{HH}	H3	ab	MET	8.98	2.29	51	2.67	10.5	10.5	10.5	7.83
Fab	H3	ab	TYR	8.97	2.5	1725	2.5	10.5	10.5	10.5	8
Fab	H1	ab	TRP	8.96	1.99	139	3.98	10.04	10.5	10.5	6.52
scFv	H3	ab	TYR	8.95	2.56	127	2.5	10.5	10.5	10.5	8
V_{HH}	H2	ab	HIS	8.89	2.37	15	4.49	10.5	10.5	10.5	6.01

scFv	H1	ab	ASP	8.82	2.08	14	4.77	10.05	10.5	10.5	5.73
Fab	H2	ab	ARG	8.82	2.48	107	2.63	10.34	10.5	10.5	7.87
scFv	L2	ab	ARG	8.82	2.8	7	4.69	10.5	10.5	10.5	5.81
Fab	H2	ab	TRP	8.81	2.2	101	3.15	10.31	10.5	10.5	7.35
scFv	L1	ab	ARG	8.78	2.13	11	5.26	10.11	10.5	10.5	5.24
scFv	H3	ab	THR	8.77	2.01	26	4.53	10.01	10.5	10.5	5.97
scFv	H3	ab	GLU	8.75	2.14	18	4.74	10.5	10.5	10.5	5.76
Fab	L2	ab	MET	8.64	2.14	6	6.08	9.23	10.5	10.5	4.42
scFv	L2	ab	PHE	8.62	1.49	6	7.26	8.02	10.5	10.5	3.24
V_{HH}	H3	ab	LEU	8.61	2.66	196	2.86	10.5	10.5	10.5	7.64
scFv	L3	ab	ILE	8.6	3.25	7	3.62	10.5	10.5	10.5	6.88
scFv	L1	ab	TYR	8.58	2.37	45	2.5	10.09	10.5	10.5	8
Fab	H2	ab	TYR	8.56	2.52	501	2.5	9.92	10.5	10.5	8
Fab	H2	ab	GLN	8.53	2.84	25	3.12	10.49	10.5	10.5	7.38
scFv	H2	ab	TRP	8.51	1.46	4	6.7	8.63	10.08	6.7	3.38
Fab	H3	ab	TRP	8.51	2.63	323	2.5	10.21	10.5	10.5	8
V_{HH}	H3	ab	ARG	8.5	2.62	329	2.5	10.25	10.5	10.5	8
V_{HH}	H2	ab	TRP	8.49	2.58	70	2.94	10.18	10.5	10.5	7.56
V_{HH}	H3	ab	ILE	8.43	2.68	117	2.6	10.36	10.5	10.5	7.9
scFv	H2	ab	TYR	8.34	2.41	40	2.75	9.23	10.5	10.5	7.75
Fab	L1	ab	TYR	8.23	2.68	593	2.5	9.67	10.5	10.5	8
scFv	L2	ab	TRP	8.2	3.15	5	4.71	10.5	10.5	10.5	5.79
V_{HH}	H3	ab	PHE	8.18	2.82	136	2.52	10.38	10.5	10.5	7.98
V_{HH}	H2	ab	PHE	8.09	2.54	20	3.68	9.12	10.5	10.5	6.82
Fab	L1	ab	TRP	7.81	2.56	41	2.89	8.19	10.5	10.5	7.61
V_{HH}	H2	ab	TYR	7.74	2.63	52	3.12	7.78	10.5	10.5	7.38
scFv	H1	ab	ARG	7.63	3.75	5	2.64	9.95	10.5	10.5	7.86
scFv	H3	ab	TRP	7.45	2.63	16	2.87	6.74	10.5	10.5	7.63
scFv	L2	ab	HIS	7.43	0.57	2	7.03	7.43	7.83	7.03	0.8

V_{HH}	H1	ab	TRP	7.39	2.62	16	2.5	6.65	10.5	10.5	8
Fab	H2	ab	PHE	7.2	3.13	91	2.6	8.69	10.5	10.5	7.9
V_{HH}	H3	b	TRP	7.09	3	118	2.5	7.09	10.5	10.5	8
scFv	H2	b	GLU	5.65	3.4	8	2.5	4.77	10.5	10.5	8
scFv	L1	b	TRP	3.91	NA	1	3.91	3.91	3.91	3.91	0

Table S6. Summary statistics for use of concavity (Å) by antigen at deepest and on average in antibody-antigen interfaces

Concavity	Ab Type	ANOVA Group†	Mean	SD	n	Min	Med	Max	Mode	Range
average	V_{HH}	a	10.12	0.2	309	9.26	10.17	10.5	10.19	1.24
average	Fab	b	9.92	0.35	847	7.68	9.99	10.5	10.2	2.82
average	scFv	b	9.86	0.41	64	8.38	9.98	10.49	10.1	2.11
deepest	V_{HH}	a	3.88	1.34	309	2.5	3.46	10.5	3.08	8
deepest	scFv	ab	3.74	1.34	64	2.5	3.46	10.33	2.5	7.83
deepest	Fab	b	3.44	1.02	847	2.5	3.17	10.5	2.5	8

† ANOVA was conducted separately for “average” and “deepest” use of concavity

Table S7. Summary statistics and ANOVA analysis for antigen amino acid use of concavity in antibody surfaces.

Ab Type	Amino Acid	ANOVA Group	Mean	SD	n	Min	Med	Max	Mode	Range
scFv	TRP	a	10.37	0.27	**22**	9.32	10.5	10.5	10.5	1.18
Fab	CYS	a	9.98	1.12	**305**	4.52	10.5	10.5	10.5	5.98
scFv	VAL	a	9.97	1.05	**67**	5.77	10.5	10.5	10.5	4.73
V_{HH}	CYS	a	9.93	1.46	**93**	3.53	10.5	10.5	10.5	6.97
V_{HH}	GLY	a	9.73	1.58	**449**	2.54	10.5	10.5	10.5	7.96
V_{HH}	VAL	a	9.62	1.73	**359**	2.7	10.5	10.5	10.5	7.8
V_{HH}	ALA	a	9.57	1.77	**333**	2.89	10.5	10.5	10.5	7.61
V_{HH}	PRO	a	9.56	1.65	**292**	3.48	10.5	10.5	10.5	7.02
V_{HH}	LEU	a	9.46	1.92	**471**	2.67	10.5	10.5	10.5	7.83
V_{HH}	ILE	a	9.45	1.88	**314**	2.75	10.5	10.5	10.5	7.75
V_{HH}	SER	a	9.44	1.95	**499**	2.92	10.5	10.5	10.5	7.58
scFv	GLN	ab	9.4	2.09	**49**	3.92	10.5	10.5	10.5	6.58
Fab	VAL	ab	9.38	1.99	**824**	2.78	10.5	10.5	10.5	7.72
Fab	ILE	ab	9.37	2.04	**782**	2.71	10.5	10.5	10.5	7.79
Fab	THR	ab	9.35	2.08	**1303**	2.54	10.5	10.5	10.5	7.96
V_{HH}	THR	ab	9.35	1.98	**416**	2.53	10.5	10.5	10.5	7.97
V_{HH}	LYS	ab	9.34	1.95	**379**	2.67	10.5	10.5	10.5	7.83
scFv	PHE	ab	9.34	2.05	**46**	3.22	10.38	10.5	10.5	7.28
Fab	SER	ab	9.33	1.99	**1384**	2.59	10.5	10.5	10.5	7.91
Fab	MET	ab	9.32	2.08	**289**	2.5	10.5	10.5	10.5	8
scFv	LEU	ab	9.29	1.96	**94**	3.19	10.5	10.5	10.5	7.31
Fab	GLY	ab	9.27	2.09	**1443**	2.64	10.5	10.5	10.5	7.86
V_{HH}	PHE	ab	9.27	2.24	**322**	2.5	10.5	10.5	10.5	8
scFv	PRO	ab	9.27	2.07	**59**	3.01	10.5	10.5	10.5	7.49
scFv	HIS	ab	9.25	2.1	**34**	3.45	10.47	10.5	10.5	7.05

scFv	SER	ab	9.21	2.03	**95**	2.5	10.5	10.5	10.5	8
V_{HH}	ASN	ab	9.2	2.16	**434**	2.65	10.5	10.5	10.5	7.85
scFv	ALA	ab	9.18	2.4	**64**	3.22	10.5	10.5	10.5	7.28
scFv	GLY	ab	9.17	2.22	**95**	3.04	10.5	10.5	10.5	7.46
Fab	LEU	ab	9.13	2.18	**1188**	2.66	10.5	10.5	10.5	7.84
Fab	PRO	ab	9.1	2.2	**912**	2.68	10.48	10.5	10.5	7.82
V_{HH}	MET	ab	9.1	2.32	**107**	2.77	10.5	10.5	10.5	7.73
Fab	TRP	ab	9.08	2.33	**372**	2.6	10.5	10.5	10.5	7.9
V_{HH}	TYR	ab	9.06	2.2	**438**	2.61	10.5	10.5	10.5	7.89
Fab	ALA	ab	9.03	2.2	**807**	2.96	10.5	10.5	10.5	7.54
V_{HH}	GLN	ab	9.03	2.32	**352**	2.59	10.5	10.5	10.5	7.91
V_{HH}	ASP	ab	9.02	2.37	**457**	2.52	10.5	10.5	10.5	7.98
Fab	HIS	ab	8.99	2.36	**487**	2.5	10.5	10.5	10.5	8
Fab	PHE	ab	8.96	2.3	**730**	2.62	10.41	10.5	10.5	7.88
Fab	TYR	ab	8.96	2.34	**992**	2.5	10.48	10.5	10.5	8
V_{HH}	ARG	ab	8.96	2.11	**400**	2.52	10.27	10.5	10.5	7.98
Fab	ASN	ab	8.95	2.34	**1324**	2.52	10.5	10.5	10.5	7.98
Fab	ASP	ab	8.87	2.33	**1287**	2.5	10.44	10.5	10.5	8
V_{HH}	TRP	ab	8.87	2.47	**125**	2.5	10.5	10.5	10.5	8
scFv	ILE	ab	8.87	2.57	**37**	3.02	10.5	10.5	10.5	7.48
V_{HH}	HIS	ab	8.84	2.24	**167**	3.08	10.16	10.5	10.5	7.42
Fab	GLN	ab	8.8	2.51	**1026**	2.5	10.45	10.5	10.5	8
scFv	ASN	ab	8.8	2.68	**111**	2.5	10.5	10.5	10.5	8
V_{HH}	GLU	ab	8.78	2.51	**527**	2.5	10.5	10.5	10.5	8
scFv	THR	ab	8.78	2.37	**86**	3.34	10.42	10.5	10.5	7.16
scFv	MET	ab	8.76	3.46	**9**	2.61	10.5	10.5	10.5	7.89
Fab	GLU	ab	8.75	2.52	**1334**	2.5	10.48	10.5	10.5	8
scFv	LYS	ab	8.75	2.45	**113**	3.45	10.44	10.5	10.5	7.05
scFv	CYS	ab	8.72	2.38	**21**	4.11	10.47	10.5	10.5	6.39

Fab	LYS	ab	8.64	2.6	**1416**	2.5	10.35	10.5	10.5	8
scFv	TYR	ab	8.62	2.57	**72**	2.74	10.22	10.5	10.5	7.76
scFv	ASP	ab	8.44	2.77	**84**	2.72	10.39	10.5	10.5	7.78
Fab	ARG	b	8.23	2.81	**1238**	2.5	9.97	10.5	10.5	8
scFv	GLU	b	8.14	2.59	**120**	2.5	9.38	10.5	10.5	8
scFv	ARG	b	7.96	2.77	**70**	2.57	8.63	10.5	10.5	7.93

Table S8. Summary statistics and ANOVA analysis of interatomic interactions utilised per 100Å^2 for Fab, scFv and V_{HH} antibodies.

Ab Type	Interaction Type	ANOVA Group	Mean	SD	n	Min	Med	Max	Mode	Range
V_{HH}	Proximal	a	27.58	4.66	309	8.19	27.84	37.43	28.05	29.24
scFv	Proximal	ab	27.18	4.63	64	7.57	27.28	36.18	22.38	28.62
Fab	Proximal	b	27.1	4.46	847	6.81	26.83	80.45	26.73	73.64
V_{HH}	Hydrophobic	c	1.98	0.91	309	0	1.9	5.24	0	5.24
scFv	Hydrophobic	c	1.86	0.66	64	0	1.87	3.31	1.23	3.31
Fab	Hydrophobic	c	1.79	0.83	847	0	1.66	4.71	0	4.71
Fab	VDWClash	d	0.8	0.31	847	0	0.76	3.2	0.79	3.2
scFv	Polar	d	0.8	0.22	64	0.21	0.78	1.24	0.48	1.03
Fab	Polar	d	0.79	0.29	847	0	0.77	4.11	0	4.11
V_{HH}	VDWClash	d	0.77	0.3	309	0.11	0.74	2.1	0.68	1.99
V_{HH}	Polar	d	0.76	0.28	309	0	0.75	1.53	0	1.53
scFv	VDWClash	de	0.73	0.27	64	0	0.7	1.28	0.32	1.28
scFv	WeakPolar	def	0.63	0.25	64	0	0.64	1.22	0.27	1.22
V_{HH}	WeakPolar	def	0.62	0.25	309	0	0.59	1.58	0	1.58
Fab	WeakPolar	def	0.61	0.24	847	0	0.6	3.05	0	3.05
scFv	Hbond	def	0.57	0.22	64	0	0.55	1.14	0.32	1.14
Fab	Hbond	def	0.56	0.23	847	0	0.56	2.13	0	2.13
V_{HH}	Hbond	def	0.55	0.23	309	0	0.56	1.19	0	1.19
scFv	WeakHbond	defg	0.5	0.19	64	0	0.48	0.99	0	0.99
scFv	VDW	defg	0.44	0.21	64	0	0.44	1.02	0.21	1.02
Fab	WeakHbond	defg	0.43	0.19	847	0	0.43	1.83	0	1.83
V_{HH}	WeakHbond	defg	0.43	0.19	309	0	0.42	1.17	0	1.17
Fab	VDW	defg	0.42	0.18	847	0	0.41	1.68	0	1.68
V_{HH}	VDW	defg	0.41	0.2	309	0	0.38	1.05	0	1.05
V_{HH}	Ionic	defg	0.28	0.3	309	0	0.2	2.23	0	2.23

scFv	Ionic	defg	0.26	0.27	64	0	0.2	0.95	0	0.95
Fab	Ionic	efg	0.26	0.26	847	0	0.2	1.62	0	1.62
V_{HH}	Aromatic	efg	0.25	0.37	309	0	0.09	2.02	0	2.02
Fab	Aromatic	efg	0.23	0.34	847	0	0.07	2.45	0	2.45
Fab	Carbon.PI	efg	0.23	0.17	847	0	0.21	1.11	0	1.11
V_{HH}	Carbon.PI	efg	0.23	0.18	309	0	0.19	0.87	0	0.87
scFv	Carbon.PI	efg	0.22	0.12	64	0	0.21	0.58	0	0.58
scFv	Aromatic	efg	0.18	0.3	64	0	0	1.22	0	1.22
Fab	PI.PI	fg	0.16	0.22	847	0	0.1	1.72	0	1.72
V_{HH}	PI.PI	fg	0.16	0.22	309	0	0.11	1.47	0	1.47
scFv	PI.PI	fg	0.13	0.21	64	0	0	1.05	0	1.05
Fab	Cation.PI	g	0.04	0.08	847	0	0	0.67	0	0.67
Fab	Donor.PI	g	0.04	0.05	847	0	0	0.29	0	0.29
scFv	Donor.PI	g	0.04	0.05	64	0	0	0.19	0	0.19
V_{HH}	Carbonyl	g	0.04	0.06	309	0	0	0.3	0	0.3
V_{HH}	Cation.PI	g	0.04	0.08	309	0	0	0.44	0	0.44
Fab	Amide.Amide	g	0.03	0.05	847	0	0	0.34	0	0.34
Fab	Amide.Ring	g	0.03	0.05	847	0	0	0.34	0	0.34
Fab	Carbonyl	g	0.03	0.04	847	0	0	0.22	0	0.22
scFv	Amide.Ring	g	0.03	0.04	64	0	0	0.14	0	0.14
scFv	Carbonyl	g	0.03	0.05	64	0	0	0.26	0	0.26
scFv	Cation.PI	g	0.03	0.07	64	0	0	0.41	0	0.41
V_{HH}	Amide.Amide	g	0.03	0.05	309	0	0	0.33	0	0.33
V_{HH}	Amide.Ring	g	0.03	0.05	309	0	0	0.32	0	0.32
V_{HH}	Donor.PI	g	0.03	0.05	309	0	0	0.37	0	0.37
scFv	Amide.Amide	g	0.02	0.04	64	0	0	0.19	0	0.19
Fab	MetalSulphur.PI	g	0.01	0.04	847	0	0	0.29	0	0.29
V_{HH}	MetalSulphur.PI	g	0.01	0.04	309	0	0	0.33	0	0.33
Fab	Clash	g	0	0	847	0	0	0	0	0

Fab	Covalent	g	0	0	847	0	0	0.1	0	0.1
Fab	Halogen	g	0	0	847	0	0	0	0	0
Fab	Halogen.PI	g	0	0	847	0	0	0	0	0
Fab	Metal	g	0	0	847	0	0	0	0	0
scFv	Clash	g	0	0	64	0	0	0	0	0
scFv	Covalent	g	0	0	64	0	0	0	0	0
scFv	Halogen	g	0	0	64	0	0	0	0	0
scFv	Halogen.PI	g	0	0	64	0	0	0	0	0
scFv	Metal	g	0	0	64	0	0	0	0	0
scFv	MetalSulphur.PI	g	0	0.02	64	0	0	0.11	0	0.11
V_{HH}	Clash	g	0	0	309	0	0	0	0	0
V_{HH}	Covalent	g	0	0	309	0	0	0.05	0	0.05
V_{HH}	Halogen	g	0	0	309	0	0	0	0	0
V_{HH}	Halogen.PI	g	0	0	309	0	0	0	0	0
V_{HH}	Metal	g	0	0	309	0	0	0	0	0
